# A Dexterous Robotic System for Autonomous Debridement of Osteolytic Bone Lesions in Confined Spaces: Human Cadaver Studies

**DOI:** 10.1109/tro.2021.3091283

**Published:** 2021-07-21

**Authors:** Shahriar Sefati, Rachel Hegeman, Iulian Iordachita, Russell H. Taylor, Mehran Armand

**Affiliations:** Laboratory for Computational Sensing and Robotics, Johns Hopkins University, Baltimore, MD 21218 USA; Laboratory for Computational Sensing and Robotics, Johns Hopkins University, Baltimore, MD 21218 USA; Laboratory for Computational Sensing and Robotics, Johns Hopkins University, Baltimore, MD 21218 USA; Laboratory for Computational Sensing and Robotics, Johns Hopkins University, Baltimore, MD 21218 USA; Department of Orthopedic Surgery, The Johns Hopkins Medical School, Baltimore, MD 21205 USA; Laboratory for Computational Sensing and Robotics, Johns Hopkins University, Baltimore, MD 21218 USA

**Keywords:** Autonomy in medical robotics, dexterous continuum manipulator (CM), minimally invasive surgery, orthopedic surgery

## Abstract

This article presents a dexterous robotic system for autonomous debridement of osteolytic bone lesions in confined spaces. The proposed system is distinguished from the state-of-the-art orthopedics systems because it combines a rigid-link robot with a continuum manipulator (CM) that enhances reach in difficult-to-access spaces often encountered in surgery. The CM is equipped with flexible debriding instruments and fiber Bragg grating sensors. The surgeon plans on the patient’s preoperative computed tomography and the robotic system performs the task autonomously under the surgeon’s supervision. An optimization-based controller generates control commands on the fly to execute the task while satisfying physical and safety constraints. The system design and controller are discussed and extensive simulation, phantom and human cadaver experiments are carried out to evaluate the performance, workspace, and dexterity in confined spaces. Mean and standard deviation of target placement are 0.5 and 0.18 mm, and the robotic system covers 91% of the workspace behind an acetabular implant in treatment of hip osteolysis, compared to the 54% that is achieved by conventional rigid tools.

## I. Introduction

Robotic technology is one of the fastest growing sectors within the healthcare industry [1]. Over the past decade, robots have augmented nearly two million surgical operations worldwide [2]. From tele-operated (direct) control to cooperative (shared) control and to autonomous control, these robotic systems have exhibited different degrees of autonomy [3]. Even though autonomy has been a familiar concept in surgical robotics since the early days [2], more attempts have been made recently to provide clearer definitions of different levels of autonomy in medical robots, as well as the associated regulatory and ethical considerations [1], [3]–[5], similar to other fields, such as autonomous self-driving cars (e.g., [6]).

Orthopedics was one of the earliest medical applications for deployment of autonomous surgical robotics in the operating room [2]. ROBODOC (originally Integrated Surgical Systems, Inc., Sacramento, CA, USA, currently TSolution One by Curexo Technology Corporation, Fremont, CA, USA) was the first commercially available active medical robot in any discipline, which allowed precision planning and autonomous milling for the femoral component in total hip arthroplasty (THA) and later total knee arthroplasty (TKA) [7]. The iBlock robotic cutting guide (OMNIlife Science, East Taunton, MA, USA) is another example of autonomous control in orthopedic surgical robotic systems [8]. The MAKO robotic arm (Stryker, Mahwah, NJ, USA) incorporates a shared control scheme for THA and TKA, which acts as a hand-held robotic device enforcing cutting boundary guides on the surgeon’s hands via virtual fixtures (VF) [8]. Other examples of orthopedic surgical robots with a shared control scheme include Navio PFS (Smith and Nephew, Memphis, TN, USA) and ROSA (ROSA Knee Robot, Zimmer Biomet, Warsaw, IN, USA), among others found in [2] and [7]. All of the aforementioned systems depend on rigid-link robots to perform the surgical tasks.

Over the past decades, minimally invasive surgery (MIS) has become an appealing trend in robotic surgery [9]–[11] due to the many advantages it offers, such as reducing patient discomfort. Even though robotics has generally improved performance in MIS, adaptation to confined, difficult-to-access surgical sites have been limited [12]. Continuum manipulators (CMs) address this by providing enhanced dexterity, flexibility, and reach [13]–[17]. An extensive review on the use of CMs across surgical applications, including neurosurgery, otolaryngology, cardiac, vascular, abdominal, and urology, can be found in [12]. In comparison to these surgical domains, orthopedic surgeries involve interactions of instruments with hard tissues and bone, resulting in significantly higher contact forces. As such, despite all the benefits that CMs offer compared to conventional rigid-link robotic manipulators, their use in orthopedic interventions has not yet been apparent.

**Fig. 1.**
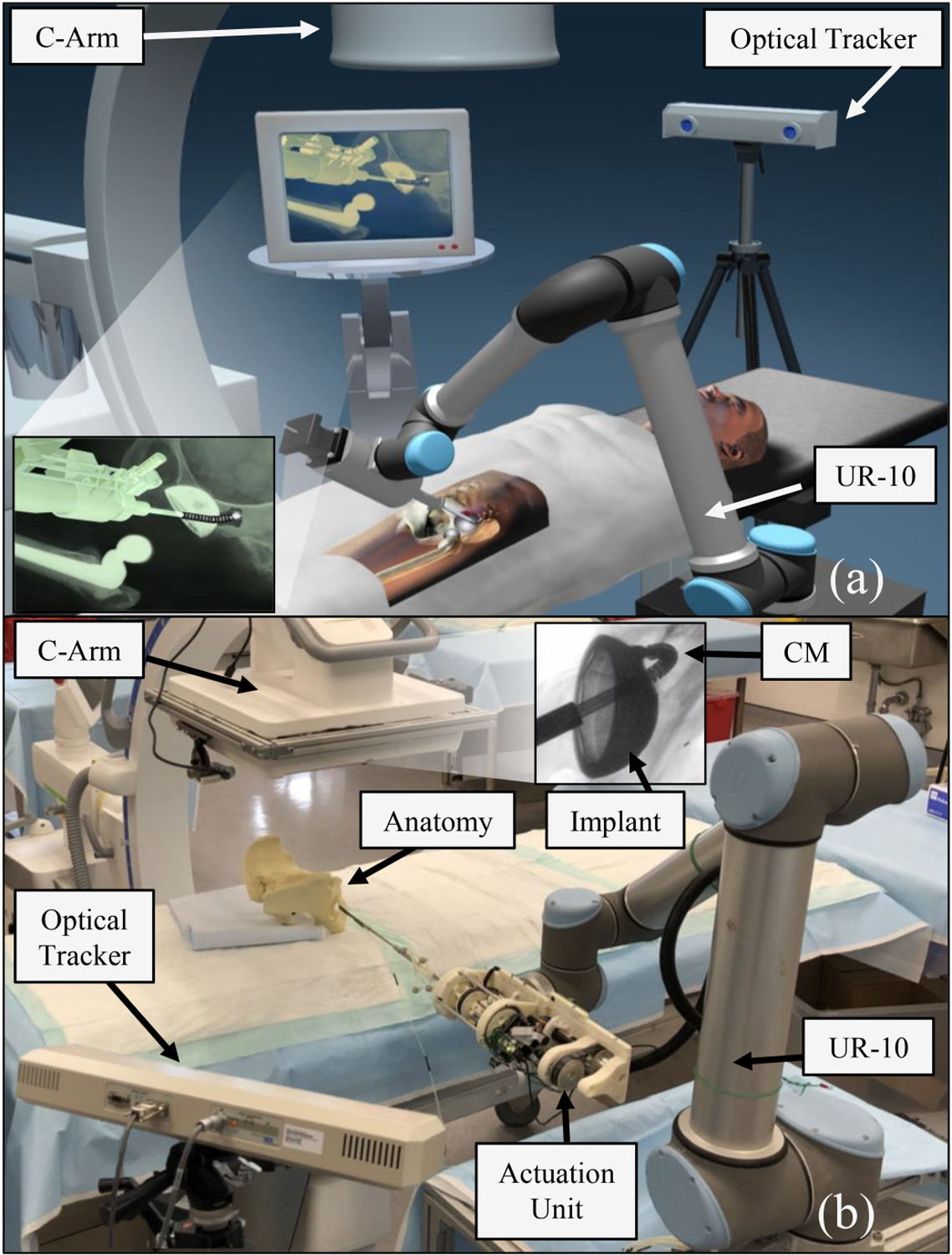
Surgical workstation. (a) Envisioned system. (b) Developed system deployed in the operating room.

We present a surgical workstation containing a dexterous and redundant robotic system for the autonomous removal of bone lesions in confined spaces by combining a conventional rigid-link robot for general positioning and a CM developed specifically for orthopedic applications [18]–[20]. The CM design allows for a great degree of flexibility and dexterity for enhanced maneuvers in confined spaces, yet sufficient rigidity during interactions with bone or hardened tissues. The CM is equipped with advanced FBG sensing technology [21] for real-time shape sensing and tip position estimation with submillimeter accuracy, and flexible instruments to perform various debridement tasks while following the CM’s nonconstant curvature shape during articulation. The surgical workstation additionally contains a C-arm for optional intraoperative intermittent X-rays for navigation. An optimization-based multiobjective controller framework is implemented to facilitate incorporation of various safety and surgical constraints (i.e., VFs) applicable to the surgery. The controller generates joint-level velocity commands on the fly to drive the system autonomously on desired trajectories specified by the surgeon while satisfying the safety constraints. An overview of our envisioned and developed robot-assisted surgical workstations is shown in Fig. 1.

An immediate orthopedic surgery candidate that could benefit from the dexterity of the proposed system is the treatment of pelvic osteolysis during the hip revision surgery [22]. After THA, the wear of the polyethylene liner leads to the formation of polyethylene particles that cause macrophage activation and consequently degradation of the bone surrounding the acetabular implant. Conventional treatment of osteolysis involves insertion of rigid instruments through the screw hole of the acetabular implant. However, a review of the literature indicates that on average, less than 50% of the lesion is successfully removed in this approach [23]. Additionally, there is no guarantee that only the undesired regions of bone and tissue (i.e., the lesions) are removed. Another candidate that could benefit from the developed system is the avascular necrosis (death of bone cells) of the femoral head, which causes pain and eventually leads to collapse of the subchondral bone [24], [25]. Conventional core decompression technique to enhance vascular flow using rigid tools suffers from limited accessibility and debridement of the entire lesion [26]. Our developed system, on the other hand, leverages the high flexibility of the CMs to enable access to confined regions (e.g., behind the acetabular cup or femoral head) that are difficult to reach with rigid instruments and also grants the surgeons great control over removing only the target points that they have identified as part of the lesion.

## II. Contributions

The use of CMs and flexible instruments for orthopedic applications is a challenging task. Consequently, careful design and implementation considerations are necessary to enable the effectiveness of a system for such applications. We report the development and evaluation of a dexterous robotic system using CMs for minimally invasive orthopedic interventions. The significant contributions reported in this article are as follows.

1. Development of a simulation framework for concurrent constrained motion control of the robotic system.
2. System performance and workspace evaluation in phantom and human cadaver experiments.
3. Demonstration of simultaneous reach and bone lesion debridement of difficult-to-access locations in two human anatomies namely pelvis and femur using cadaver specimens.
4. Study of the dexterity and manipulability of the developed robotic system and comparison to conventional rigid-link robots.
5. Performance comparison of model-based and model-free approaches for CM Jacobian estimation in free and constrained environments.

To the best of our knowledge, this is the first study on a surgical robotic system that incorporates the capabilities of CMs in the less and minimally invasive orthopedic interventions with debridement of hard bone.

## III. System Hardware

### A. Continuum Manipulator

**Fig. 2.**
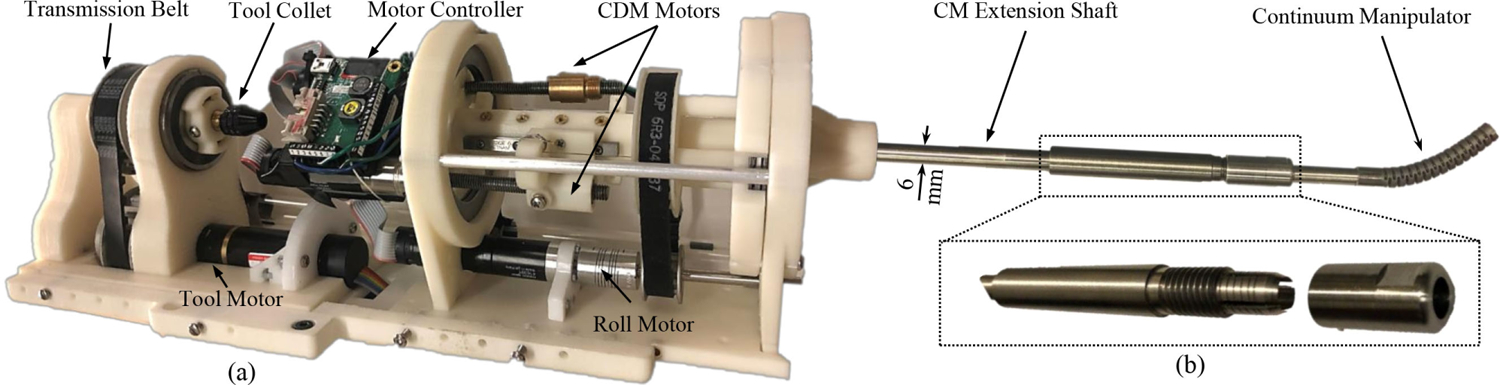
Developed actuation unit with extendable CM shaft. (a) Actuation unit. (b) Collet mechanism on the extendable shaft for quick mounting of the CM.

**Fig. 3.**
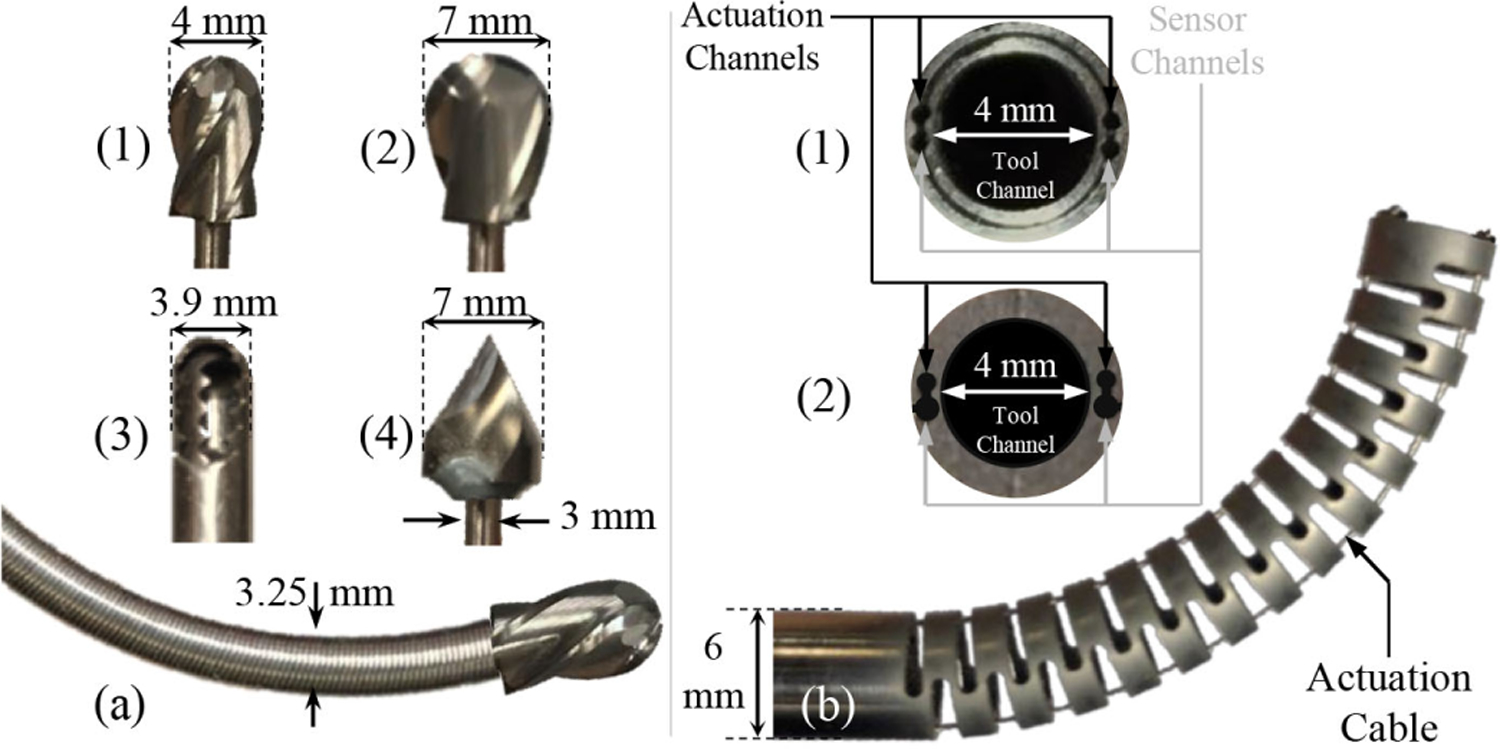
(a) Flexible instruments using a torque coil and various tool heads. (a-1) Hard-tissue milling head with four flutes. (a-2) Hard-tissue milling head with two flutes. (a-3) Soft-tissue head. (a-4) Drilling head. (b) CM and actuation cables. (b-1) First generation of CM design with two NiTi tubes. (b-2) Second generation of CM design with one NiTi rod.

The CM used in this study is developed particularly for orthopedic applications where relatively large interaction forces are present [19].The CM is cable-driven, constructed of superelastic nitinol (NiTi) tubing with several equidistant notches to achieve flexibility in the plane of articulation while remaining stiff and force-bearing in all other planes. The CM is constructed from a single NiTi tube using wire EDM on the side of the manipulator to cut the actuation cable and sensor channels in a single step [27] [see Fig.3(b-2)]. Previous iteration of the CM design with nested NiTi tubing [see Fig. 3(b-1)] is described in [18] and [19]. The inner diameter of the CM is 4 mm to allow passing of flexible debridement instruments, and the outer diameter is 6 mm so that it can fit through the screw holes of an acetabular implant. The diameters of the channels for the actuation cables and sensors are 500 and 550 *μ*m, respectively. In Fig. 3(b-2), the top and bottom CM channels are used for the actuation cables and FBG sensors, respectively. The actuation cables are stainless steel braided wires with 0.3-mm diameter.

### B. FBG Sensor

**Fig. 4.**
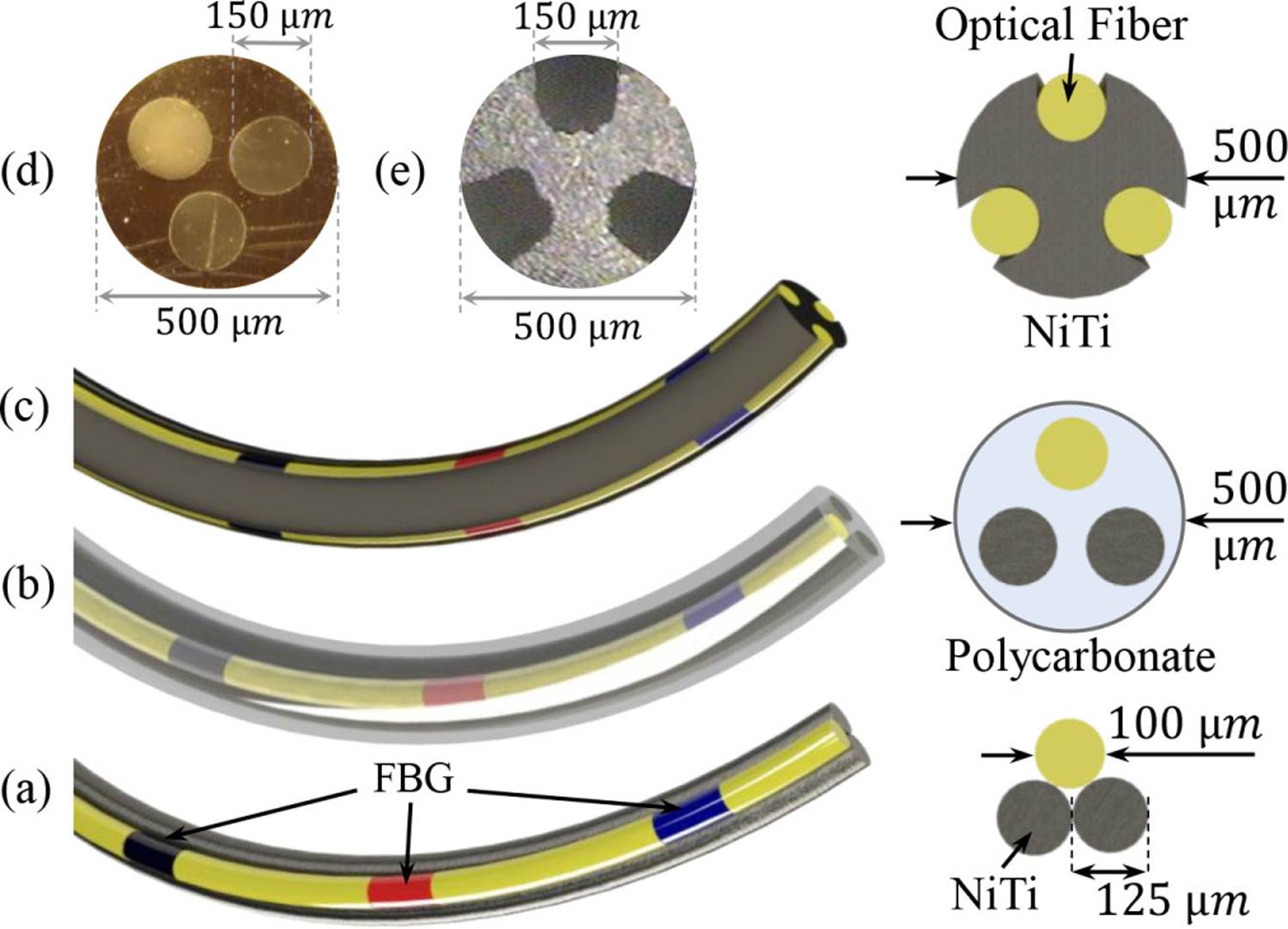
FBG sensors. (a) First generation using NiTi wires. (b) Second generation using polycarbonate tube substrate. (c) Third generation using notched NiTi substrate. (d) Polycarbonate tube cross section. (e) Notched NiTi cross section.

The FBG sensor in this study uses a flexible 500-*μ*m NiTi wire substrate with three 150-*μ*m diameter laser-engraved grooves that are radially 120° apart from each other [see Fig. 4(c) and (e)]. With direct access to the substrate grooves, three optical fibers each with three FBGs (Technica S.A, Beijing, China) are attached to the NiTi wire using epoxy glue (J-B Clear Weld Epoxy) in a triangular configuration [28], [29]. The choice of the NiTi substrate ensures structural integrity of the sensor assembly that is suited for orthopedic interventions. Previous sensor design iterations by the authors included the use of a polycarbonate tube substrate or NiTi wires with different optical fibers, sensor configurations, and number of FBG fibers [28], [30]–[33] [see Fig. 4(a) and (b)].

### C. Flexible Debridement Instruments

Various flexible instruments are custom-designed for different lesion debridement tasks, including side milling and drilling of soft [34] and hard tissue and bone [35]. All of the instruments consist of a rigid stainless steel tube (2.8-mm outer diameter) and a 35-mm length flexible torque coil (Asahi Intecc USA, Inc., Santa Ana, CA, USA) with 3.25-mm outside diameter. The torque coil provides sufficient torque to the tip of the tool for debridement tasks while it adapts to various shapes of the CM. For soft tissue debridement, a microdebrider with engaging teeth and suction capabilities was designed and studied in removing simulated soft tissue (soft gelatin phantoms) [34] [see Fig. 3(a-3)]. The main focus of this study, however, is on the harder problem of the removal of the sclerotic tissue (hard tissue). To this end, a variety of end-mill instrument heads are designed from carbide with two and four spiral flutes [see Fig. 3(a-1) and (a-2)] and with outside diameters of 9/64″, 13/64″, 15/64″, 1/4″, and 9/32″. Fig. 3(a-4) demonstrates a drilling bit instrument that could be used for curved drilling [35].

### D. Rigid-Link Robot

A 6-DOF UR10 (Universal Robots, Inc., Odense, Denmark) robot was used as the positioning rigid-link robot.

### E. Actuation Unit

Previous generations of the actuation unit can be found in [19] and [36]. The latest generation consists of two separate modular components for the CM and instrument actuation [see Fig. 2(a)] and an extendable shaft for mounting the CM using a collet mechanism [see Fig. 2(b)]. The latest generation simplifies the assembly procedure and enables the system to be used for different orthopedic surgeries with adjustable longer end-effector shaft as desired [27]. The CM actuation module contains two dc motors (RE16, Maxon Motor, Inc., Sachseln, Switzerland) with spindle drives (GP16, Maxon Motor, Inc.) equipped with load cells (Model 31 Mid, Honeywell, Inc., Charlotte, NC, USA) to actuate the CM cables. In addition, another dc motor (RE16, GP16 C, Maxon Motor, Inc.) rotates the actuation unit’s central axis (roll DOF) for CM out-of-plane motions. The instrument actuation module rotates the instruments with desired velocity by a dc motor (EC 22, GP22 C, Maxon Motor, Inc.) and a transmission and gripping mechanism. Notably, the CM actuation module provides a central channel for insertion of the instruments.

## IV. Constrained Control Framework

To increase the degrees of freedom (DOFs), the goal is to concurrently control all the system components while satisfying several safety and physical constraints associated with the surgical task. As such, a versatile optimization-based multiobjective constrained control framework is developed and built upon previous work [36], [37] to incorporate additional constraints and regularization terms beneficial in surgical scenarios. The main objective of any lesion debridement task is tracing a desired path (i.e., sets of points). Additionally, a surgical scenario typically includes additional objectives beyond only tracing a desired path. For instance, physical or safety constraints in various forms having to do with the operating room, the patient, the surgical staff, or the robotic system itself may be present. Moreover, each surgical intervention may require the robotic system to satisfy certain motion constraints specific to the surgery. Consequently, we extend our control framework to a more generalized constrained optimization problem

(1) $$\underset{\Delta q}{\text{minimize}}\;\alpha {\left\|\Delta x_{\text{obj}}-WJ_{s}\Delta q\right\|}_{2}+\beta R(\Delta q)\;\text{subject to }H\;\Delta q\leq h$$

where Δ*q* is the infinitesimal configuration space motion, Δ*x*_obj_ is the objective infinitesimal task space motion, $J_{s}\in {\mathbb{R}}^{6\times 9}$ is the system Jacobian (discussed in Section IV–D), and *q* = [*θ*_*R*1_ … *θ*_*R*6_ *θ*_*A*_ *l*_*C*1_ *l*_*C*2_] is the stack of system joint variables, including the six rigid-link robot revolute joints, the actuation unit roll joint, and the two CM actuation cable displacements. In addition, *R*(Δ*q*) could be a secondary controller task appeared in the form of a regularization or penalty term, $H\in {\mathbb{R}}^{n\times 9}$ and $h\in {\mathbb{R}}^{n\times 1}$ define *n* inequality constraints, and $W\in {\mathbb{R}}^{6\times 6}$, a diagonal weight matrix, scalar *α*, and scalar *β* (damping factor) are optional parameters for enforcing priority on specific tasks or joints. Any equality constraint can be added to the optimization problem (1) by separation to two inequality constraints.

### A. Constraints

We have incorporated several safety and physical constraints that surgeons can optionally choose to add to the constrained control framework, if desired. The general idea is that given the system joint state at current step *k*, i.e., *q*^*k*^, the Δ*q*^*k*^ command in (1) is found such that the resulting Cartesian motion *J*Δ*q*^*k*^ takes the system to a state at step *k* + 1 that satisfies a particular constraint. A detailed formulation for a variety of constraints, such as a programmable RCM, axis range VF, hyperplane VF, velocity, and joint limit constraints, can be found in Appendix A.

### B. Regularization

Secondary tasks or other constraints can be introduced to the constrained optimization problem (1) in the form of regularization or penalty (*R*(Δ*q*)). One way to interpret the regularization term is that it enforces the optimizer to prefer particular solutions over others. To improve the controller capability introduced in previous work [27], [36], [38], we provide formulation for redundancy resolution, enforcing stay near axis or pose constraints as well as a recovery strategy for infeasible optimization problems using the regularization term in Appendix B.

### C. Feedback

The system contains the following feedback components.

#### 1) FBG Sensor:

**Fig. 6.**
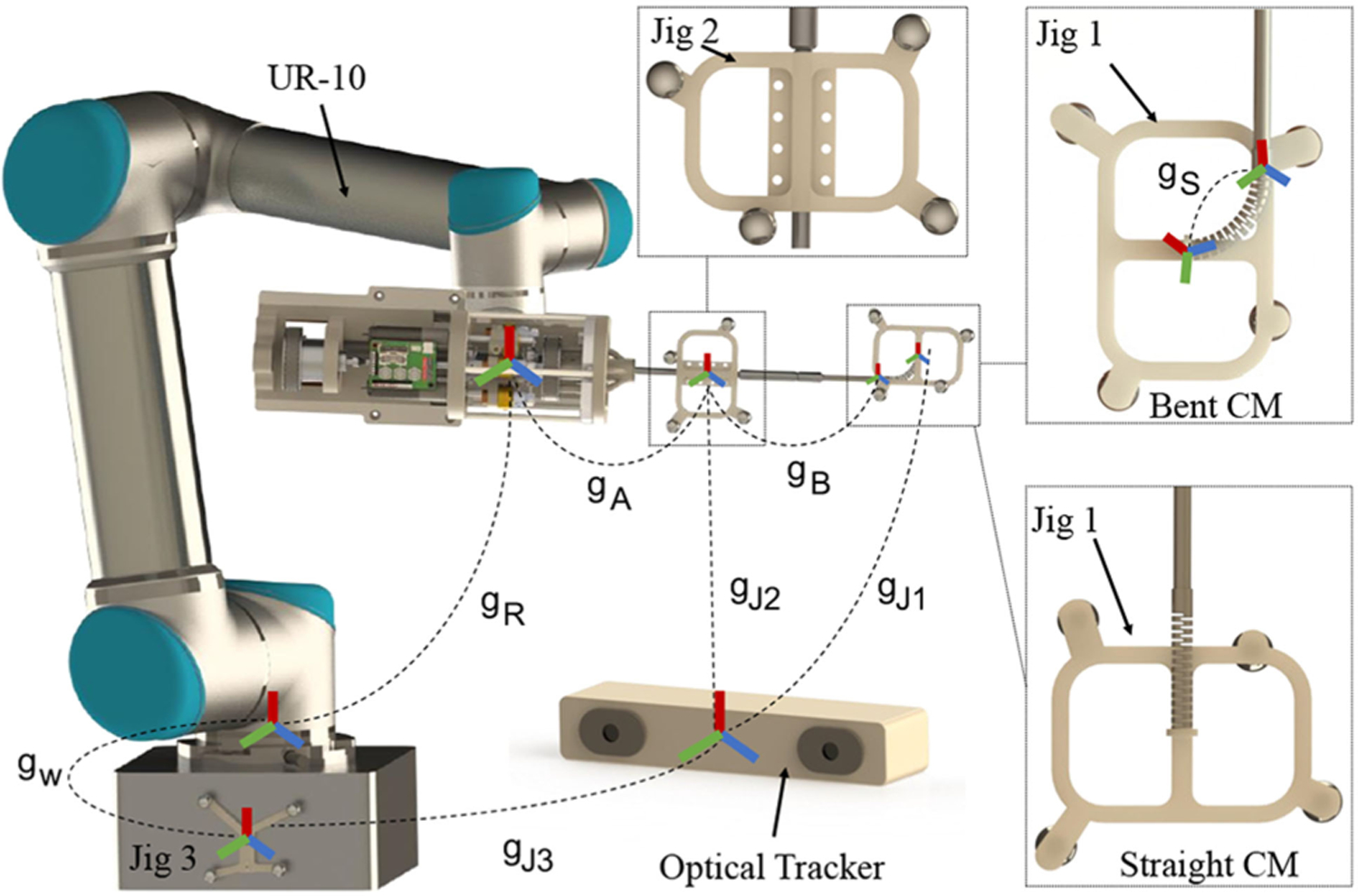
Chain of transformations in the system and the custom-designed optical tracker reflective geometries: Jig 1 mounted on the distal end of the CM, Jig 2 mounted on the end-effector shaft, and Jig 3 mounted on the base of the robot. *g_Ji_* is jig *i* observed in the optical tracker coordinate frame.

The FBG sensor is used to find the CM tip position expressed in the CM base frame. In this regard, we previously proposed a data-driven learning-based approach [39], [40] that increases the estimation accuracy and supersedes the conventional shape sensing approaches using FBGs [41]. The main idea is to find a function approximator in a supervised learning manner to estimate the unknown parameters of a model that minimizes the loss [42]. The ground-truth tip position is obtained by mounting a custom-designed optical tracker reflective jig on the CM distal end (jig 1 in Fig. 6). Preoperatively, the CM is articulated to full-bend pose a number of times and the FBG (Λ) and ground-truth tip position (*P*_gt_) are recorded. A function approximator, such as Ψ, is found by minimizing the least squares problem of. ∥ΛΨ − *P*_gt_∥ Intraoperatively, the CM tip expressed in its base frame is found via *p*_fbg_ = Ψ*λ*, where $\lambda \in {\mathbb{R}}^{9\times 1}$ is the vector of FBG readings.

#### 2) Optical Tracker:

The end-effector shaft pose could potentially be obtained from the forward kinematics; however, the uncertainties due to the backlash and friction of the actuation unit pulley transmission system can cause inaccuracies in this estimation. Consequently, an optical tracker reflective jig is mounted on the end-effector shaft for direct measurement of the pose (jig 2 in Fig. 6). To reduce measurement uncertainties, a Butterworth low-pass filter is incorporated that rejects the unwanted high-frequency components of the received signal. The system tip position expressed in the robot base frame $x_{\text{tip}}^{k}$ can be found at time step *k* by establishing appropriate coordinate frames. Given the immediate goal point at this time step $x_{\text{goal }}^{k}$ and incorporating a PD controller to tame the behavior in presence of disturbance and overshoot, $\Delta x_{\text{obj}}^{k}$ can be found by

(2) $$x_{\text{tip}}=g_{J3}^{-1}\cdot g_{J2}\cdot g_{B}\cdot p_{fbg}\;\Delta x_{\text{des}}^{k}=x_{\text{goal}}^{k}-x_{\text{tip}}^{k}\;\Delta x_{\text{obj}}^{k}=k_{p}\Delta x_{\text{des}}^{k}+k_{d}\left(\Delta x_{\text{des}}^{k}-\Delta x_{\text{des}}^{k-1}\right)$$

where *k*_*p*_ and *k*_*d*_ are the proportional and derivative gains, respectively.

### D. System Jacobian

**Fig. 7.**
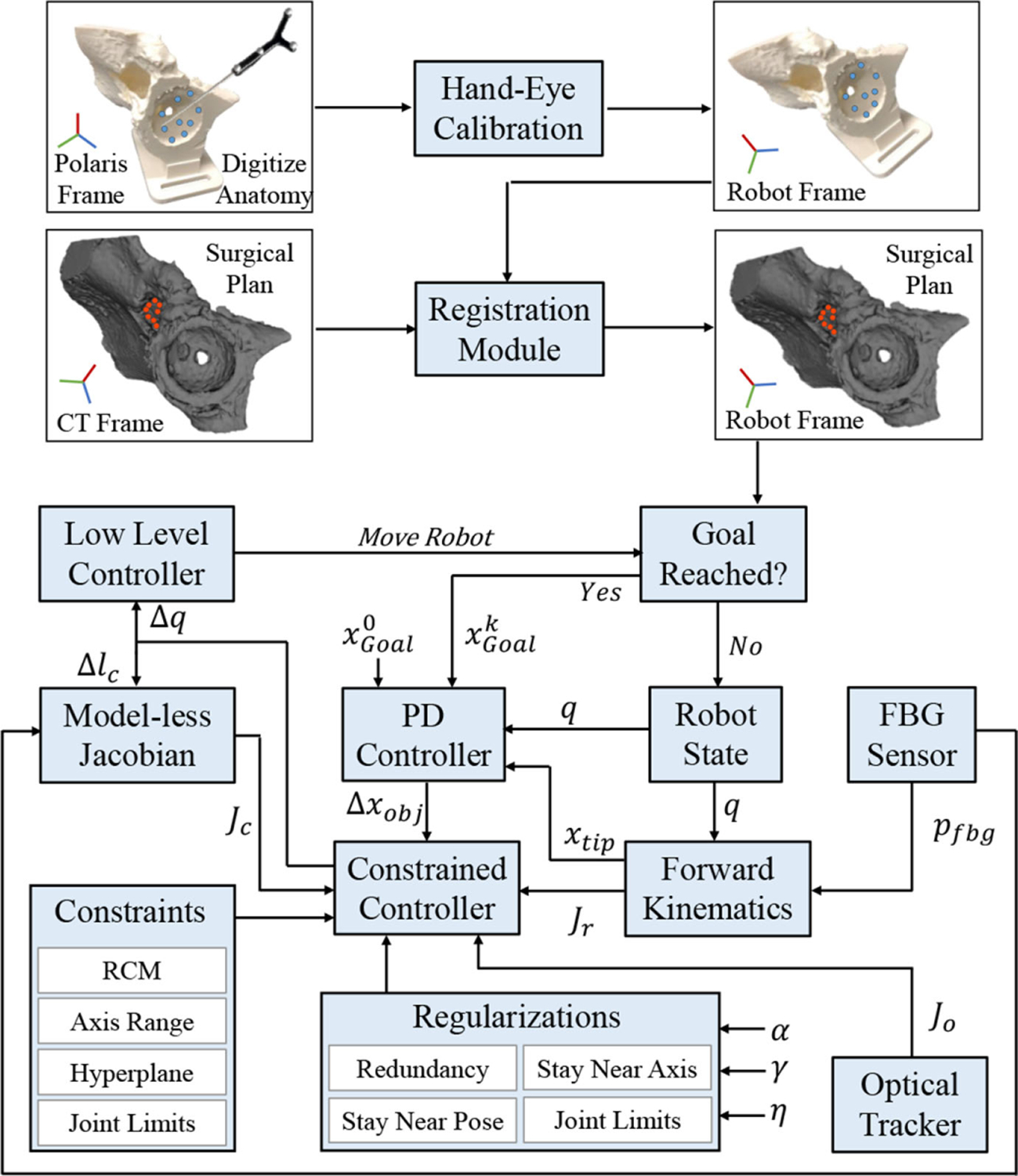
Surgical flow with planning (top) and execution (bottom) phases. Planning consists of intraoperative transformation of the digitized points on the anatomy to the robot’s coordinate frame (using the preoperative hand-eye calibration), followed by a registration step. The closed-loop control block diagram shows the execution phase with on-the-fly generation of robot commands subject to constraints and regularization.

The overall system Jacobian can be written as *J*_*s*_ = [*J*_*r*_ *J*_*a*_ *J*_*c*_], where the subscripts *r*, *a*, and *c* correspond to the UR-10 robot, the actuation unit roll joint, and the CM, respectively. We derived the geometrical linear and angular components of the Jacobian (*J*^*v*^ and *J*^*ω*^), as instructed in [43]. Since all the joints of the UR-10 and the actuation unit are revolute, for the *i*th joint (and the associated column in the Jacobian), we can write

(3) $$\{{}_{J^{\omega_{i}}=z_{i}}^{J^{v_{i}}=z_{i}\times \left(O_{r}-O_{i}\right)}$$

where *o*_*r*_ is the location of a desired point *r*, where the Jacobian is resolved at, and *z*_*i*_ and *o*_*i*_ are the axis and special position of the revolute joints expressed in the robot base coordinate frame. To find these parameters, note that for the six UR-10 joints (*J*_*r*_), the Denavit–Hartenberg parameterization of the forward kinematics (*g*_*R*_) is used, whereas for the roll joint on the actuation unit (*J*_*a*_), direct measurements from the optical tracker reflective jig (jig 2 in Fig. 6) is used to decrease uncertainties and errors due to backlash and friction. The CM Jacobian could be found using model-based data fitting or in a *model-less* manner using optimization techniques. Appendix C details the computation of *J*_*C*_ using these two approaches. Fig. 7 demonstrates the block diagram of the closed-loop control system.

## V. Software and Simulation

### A. Architecture

All the software is developed in C++ for real-time purposes. The CISST open-source multitasking library [44] is used for thread-safe parallel communication between different components of the system, i.e., the UR manipulator, the CM actuation motors, the flexible instrument motor, the FBG sensing interrogator, and the controller. The controller is implemented as a mid-level periodic task sitting in between the surgeon’s high-level control input through a graphical user interface (GUI) and the low-level controller tasks associated with the UR manipulator, and the actuation unit’s motors for the CM cables and the flexible instruments. The controller task obtains the system’s joints states and the sensory information (FBG and optical tracker) from the respective device-specific task, then sets up and solves the optimization problem and sends the commands to UR manipulator and Maxon motor low-level controllers through their designated tasks. A custom C++ interface performs low-level velocity control of the Maxon motors while the UR’s low-level control is implemented by writing a client application (URScript) and connecting to URControl using a TCP/IP socket.

### B. GUI Design

For intuitive and easy interaction of the surgeon with the system during the surgical procedure, a main GUI containing all the information about the system state, the controller status, and the surgical plan (a list of target points) is developed. Additionally, a separate panel provides the surgeon with full control over activation of their desired constraints, configuration of the parameters associated with the constraints, and execution of the surgical plan. At any point during the surgery, the surgical plan could be modified or altered altogether, and the execution of the plan could be paused or continued with the surgeon’s supervision. Other GUI tabs are also developed for direct control capability of the CM and UR-10, if desired.

### C. Visualization

**Fig. 8.**
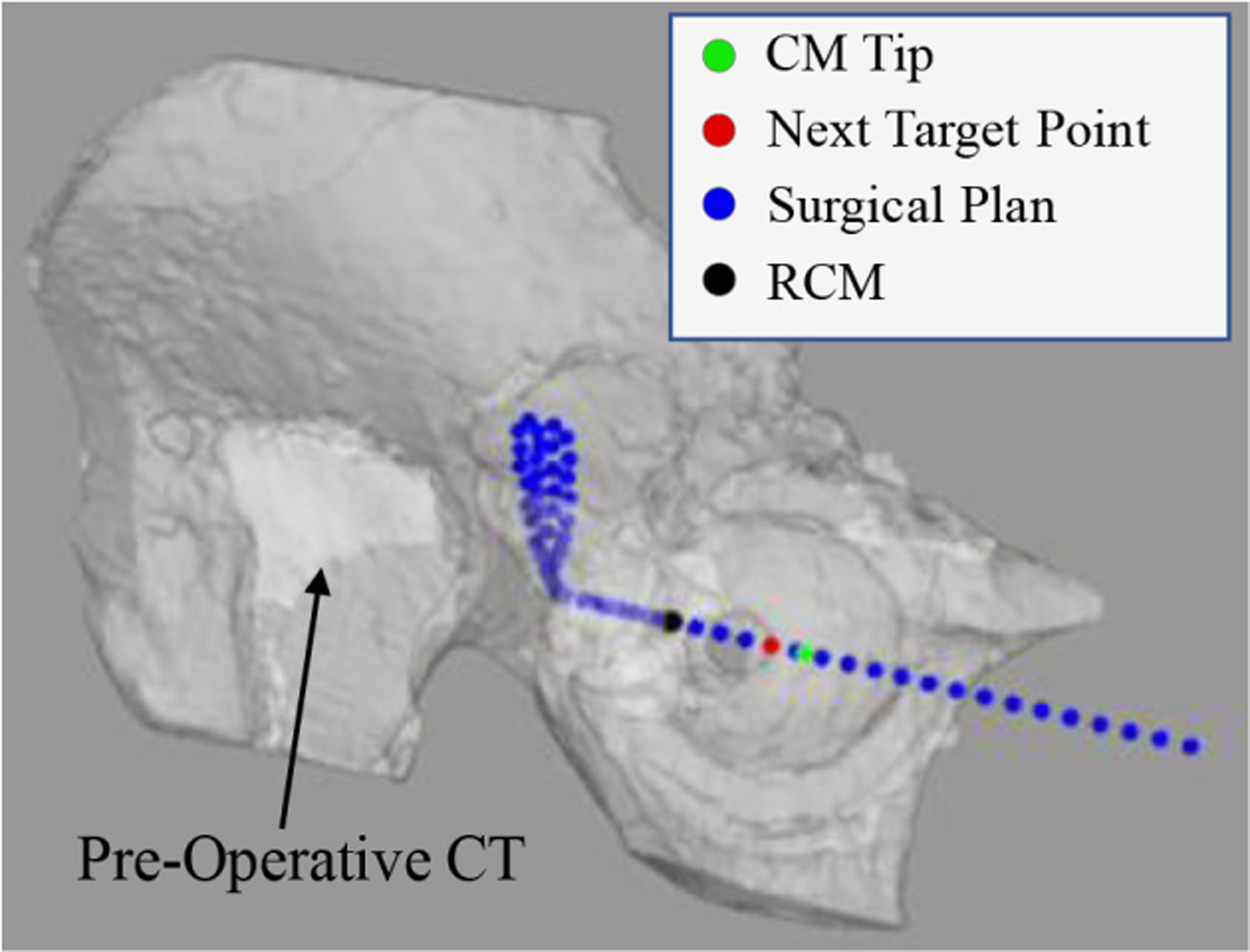
Visualization window for intraoperative navigation.

To assist the surgeon with better overview of the surgical task execution, a VTK-based [45] visualization window is developed in C++, which contains the patient’s preoperative CT overlaid with the planned target trajectory, the RCM point, and the current system tip position. The visualization is synced with the controller and the system state is demonstrated to the surgeon in real time. Fig. 8 shows this window during the execution of a surgical task.

### D. Simulation

To evaluate the proposed controller and realize the appropriate combination of constraints imposed during a surgical scenario, a complete simulation framework is developed using the robot simulator CoppeliaSim (formerly V-REP) [46]. The constraint control framework (see Section IV) is implemented in Python and the solution is communicated with the simulator using the Python remote API to update the simulation. A UR-10 robot is used as the positioning robot and the forward kinematics and the Jacobian are implemented using (3) [47]. For the simulation visualization, the CM is modeled as a 27-revolute-joint mechanism and given any actuation cable length, first the tip position (*p*) is computed from (17) and then a constrained optimization inverse kinematics is incorporated to solve for the joint angles

(4) $$\underset{\Theta_{c}}{\text{minimize}}\;{\left\|p-f\left(\Theta_{c}\right)\right\|}_{2}\;\text{subject to }\Theta_{c}\leq \Theta_{\text{max}}\;f_{x}=d\cdot \left(\overset{27}{\underset{i=1}{\sum }}\text{sin}\left(\overset{i}{\underset{j=1}{\sum }}\Theta_{j}\right)\right)\;f_{y}=d\cdot \left(\overset{27}{\underset{i=1}{\sum }}\text{cos}\left(\overset{i}{\underset{j=1}{\sum }}\Theta_{j}\right)\right)$$

where $\Theta_{c}\in {\mathbb{R}}^{27}$ is the CM joint angles, *d* = *L*_*c*_/27 is the distance between two consecutive joints, $f\left(\Theta_{c}\right):\Theta_{c}\to {\mathbb{R}}^{2}$ is the CM forward kinematics mapping from joint space to task space, and Θ_max_ is the maximum angle each joint can take and is chosen as 7.9° [18].

## VI. Preoperative Clinical Steps

### A. Calibration

Two calibration procedures must be performed prior to the surgery (not in the operating room). The first procedure is to collect the necessary sensor data to find the function approximator for the FBG sensor. For this purpose, a coordinate frame is established at the base of the CM at straight pose (enforced by a custom-designed jig) and *g*_*B*_ (jig 2 to CM’s base) is computed accordingly (see Fig. 6)

(5) $$g_{B}=g_{J2}^{-1}\cdot g_{J3}\cdot g_{S}^{-1}$$

where the rotation and translation components of, *g*_*S*_ are *I*_3×3_ and [*L*_*s*_ 0 0]^*T*^, and *L*_*s*_ = 35 mm is the length of the CM. With *g*_*B*_ known, the CM is bent to its maximum extent and the FBG data (Λ) as well as the tip ground-truth expressed with respect to the CM base coordinate frame are recorded $\left(P_{\text{gt}}=g_{B}^{-1}\cdot g_{J2}^{-1}\cdot g_{J3}\right)$. The function approximator is then estimated, as described in Section IV.

For registration purposes, an optical tracker reflective jig (jig 3) is mounted at the base of the positioning robot, which serves as the fixed (*world*) coordinate frame. To complete the chain of transformations, a hand-eye calibration procedure must take place to compute the unknown *g*_*W*_ (jig 3 to UR base) and *g*_*A*_ (UR end-effector to jig 2). The chain of transformations can be written as $g_{R}\cdot g_{A}=g_{W}\cdot g_{J1}^{-1}\cdot g_{J2}$. Taking an initial measurement when the robot is stationary, *g*_*A*_ and *g*_*W*_ can be related to one another and they can be computed subsequently by obtaining the solution to the *AX* = *XB* problem.

### B. Registration

**Fig. 9.**
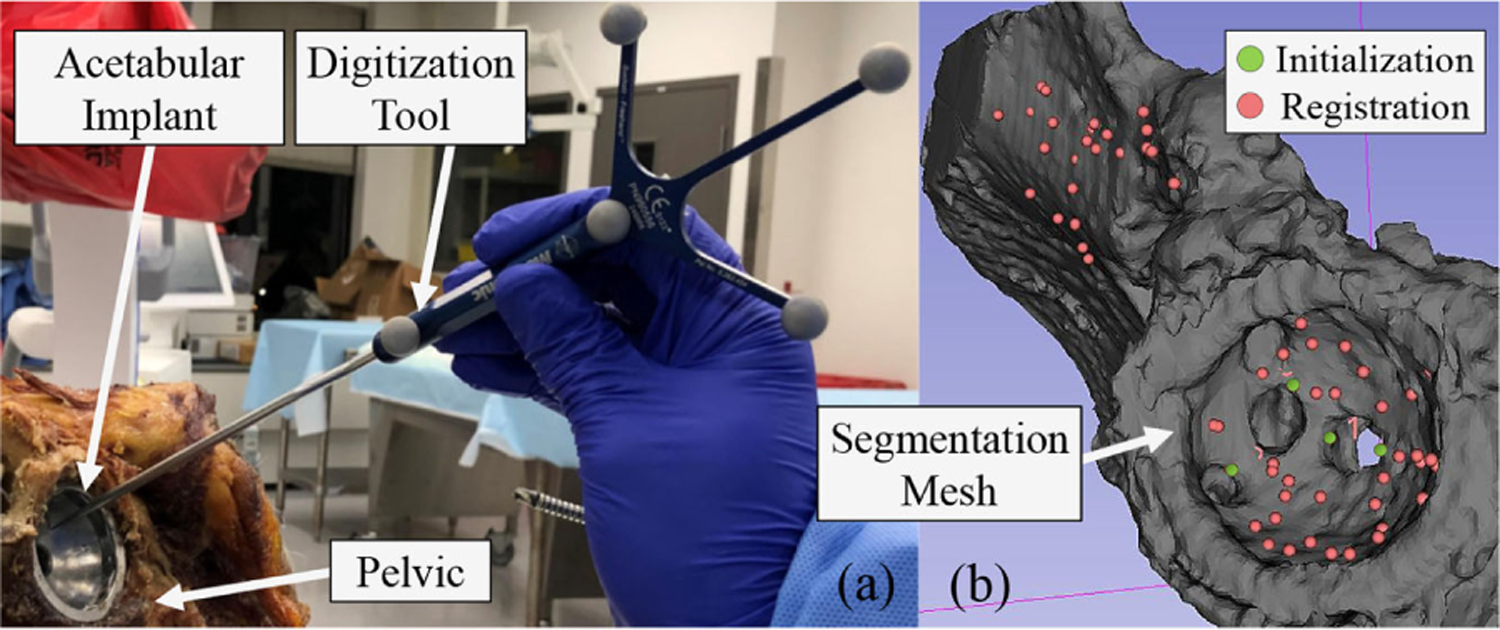
Registration procedure. (a) Digitization of the acetabular cup implant. (b) Overlayed digitized point cloud on the preoperative CT after registration.

The surgical plan is determined by the surgeon preoperatively on the patient’s CT. The CT entails additional information, such as the RCM location, axis of the screw hole, and the geometry of the lesion, all expressed in the CT coordinate frame. To register the preoperative CT model to the patient, a 3-D point cloud is collected by digitizing the surface of the acetabular implant as well as the surface of any exposed part of the surrounding bone (see Fig. 9). A digitization tool, such as the one shown in Fig. 9, can be used to form this point cloud, which is expressed in the *world* coordinate frame (jig 3 in Fig. 6). The iterative closest point approach is used to compute the registration. The first step is to compute an initial registration (*T*_0_) guess for the algorithm. This is done by selecting a couple of points on the screw holes of the implant (see Fig. 9) in the CT (*P*_ct_) and digitizing the same points on the patient with respect to the *world* coordinate frame (*P*_*w*_)

(6) $$\underset{T}{\text{minimize}}\overset{n}{\underset{i=1}{\sum }}{\left\|P_{w}^{i}-T_{i}\left(P_{\text{ct}}^{i}\right)\right\|}_{2}$$

where *T*_*i*_ ∈ *SE*(3) is the homogeneous transformation guess at the *i*th step that maps features described in the CT frame to the *world* coordinate frame. For the initialization step, *n* = 4. Next, as many points as possible are digitized from the surface of the acetabular implant and the surrounding bone. The more spread the points on the anatomy, the higher the likelihood of achieving a better registration. The software is written such that these points can be collected in a continuous (and therefore fast) motion of the digitization tool. The collected points are transformed by *T*_0_ and the closest mesh to each of these transformed points are found. A quaternion approach [48] is used to solve (6) for the next best transformations iteratively until the algorithm converges. The termination criteria are defined as *ϵ* ≤ *d*_*i*+1_/*d*_*i*_ ≤ 1, where *d*_*i*_ is the mean surface distance at step *i* and *ϵ* = 0.999 is the stopping tolerance.

## VII. Experiment Design

We ran experiments with the system in three different environments: simulation, in phantom studies on a model acetabular implant, and in cadaver studies with human specimens. The simulation environment served as a test-bed for debugging and tuning the optimization control framework, and later was used to perform a comparison of the system’s workspace and that of classical rigid surgical implements.

**Fig. 10.**
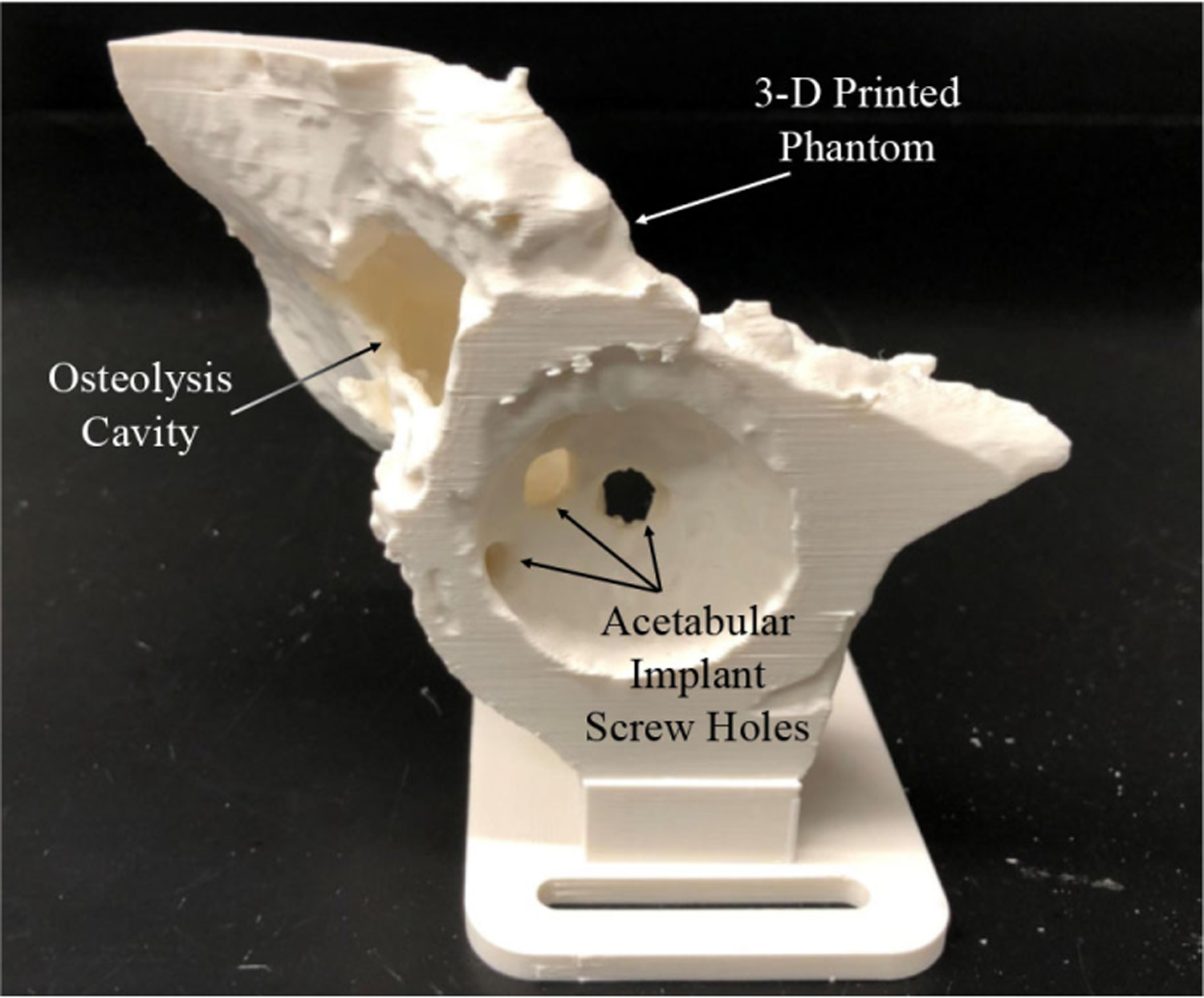
Osteolysis phantom 3-D printed from segmented CT.

In addition to simulation, phantom experiments were carried out to evaluate end-to-end system performance prior to the cadaver experiments. To accurately mimic the real surgical scenario of the less invasive treatment of pelvic osteolysis, a phantom model was obtained by performing a segmentation on a human cadaver with an outlined lesion cavity behind the acetabular cup implant created by a clinical collaborator. As shown in Fig.10, the phantom model was 3-D printed and hard sawbone phantoms with density of 15 pounds per cubic foot simulating lesions were mounted in difficult-to-reach locations behind the implant. The phantom experiments consisted of: testing and further tuning of the controller on the physical system, exploration of the outlined lesion cavity while maintaining the constraints, and debridement of the simulated sawbone phantoms.

The final sets of experiments were carried out on a pelvic human cadaver with an outlined lesion cavity created by our clinical collaborator who has previously performed conventional treatment of pelvic osteolysis behind the acetabular implant. To the best of the authors’ knowledge, this is the first human cadaver study for the less invasive treatment of pelvic osteolysis using CMs. Following were the goals of the human cadaver experiments.

1. Performing and realizing the complete preoperative clinical steps for calibration, registration, and system preparation.
2. Demonstrating the feasibility to reach difficult locations behind the cup by inserting the CM through the implant’s screw hole.
3. Comparison of the reach and workspace behind the implant by conventional rigid tools (e.g., curettes) and our developed system.
4. Drilling/milling of simulated and real bone behind the implant while concurrently controlling the system and maintaining the constraints.

It should be noted that a second cadaver experiment was also performed to demonstrate how the dexterity and flexibility of the developed system could benefit the core decompression of the femoral head osteonecrosis using the curved drilling technique. To avoid an over-lengthy results section, however, we focus the quantitative results on the pelvic osteolysis experiment and qualitatively demonstrate the capabilities of the system in the avascular necrosis procedure in Section IX due to a simpler motion control problem (only translation motion for the positioning robot).

## VIII. Evaluation Criteria and Results

We evaluated our system’s performance on the following axes: constrained workspace and reach in confined spaces; dexterity and manipulability; constrained controller performance; sensing accuracy; and planning and debridement performance.

### A. Constrained Workspace

In a typical use case of the system, the motion is subject to constraints, such as maintaining the RCM and/or limiting the end-effector’s shaft axis range. We refer to the workspace of the system with such constraints as the *constrained workspace* throughout this article.

**Fig. 11.**
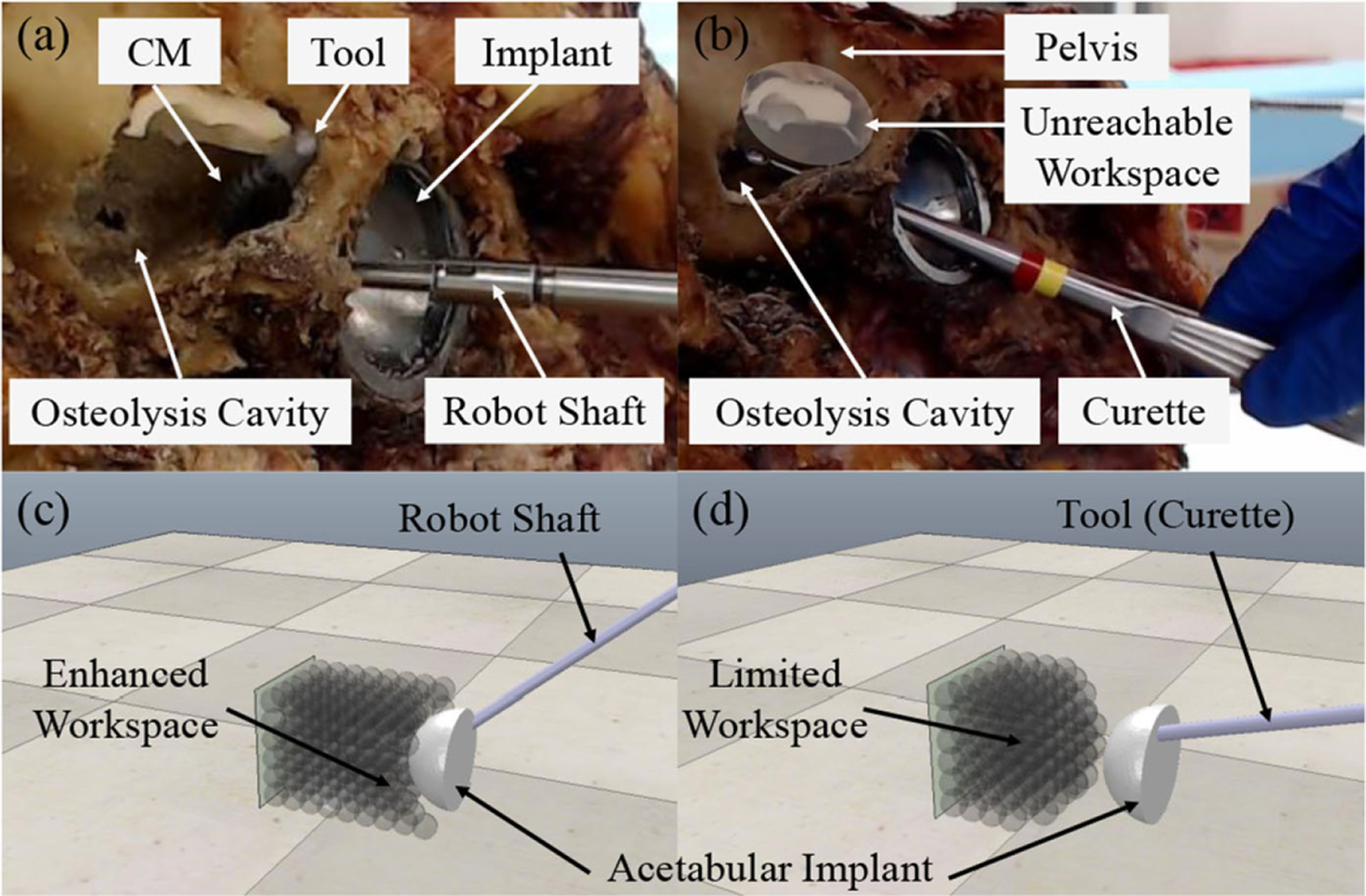
Workspace comparison of the developed dexterous robotic system and rigid tools, such as a curette in (a) and (b) during cadaver experiments, and in (c) and (d) during simulation experiments. The unreachable workspace by rigid tools behind the implant is shown in (b).

Qualitatively, the maximum reach behind the acetabular implant using a conventional rigid curette was compared with our developed system in Fig. 11(a) and (b). It should be noted that such dexterity and reach in our system was achieved by only articulating the CM and fixating the UR-10 end-effector’s axis stationary and aligning with the screw hole axis while the curette’s handle was unconstrained. Even with such a restricting constraint, points on the back surface of the implant were reachable by our system, whereas the curette’s range was quite limited.

Quantitatively, the constrained workspace of our system was compared to that of a rigid instrument, such as a curette in thorough simulation studies. An acetabular cup implant mesh with outside diameter of 50 mm, rim edge thickness of 5 mm, and screw hole diameter of 8 mm was imported in simulation. The desired target area was chosen as a cubic region extended 50-mm deep behind the acetabular implant and covering the entire back surface of the implant [see Fig. 11(c) and (d)]. Discretization of this region with 8-mm increments resulted in 264 target points. The RCM constraint was activated and the axis range VF with two different maximum allowable deviation angles (*ϕ*_*a*_ = 30°, 45°) was applied to the end-effector shaft of the robotic system as well as the handle of the rigid tool. Additionally, five hyperplane VFs bounded the target region to avoid the CM from protruding beyond this region. The robotic system and the rigid tool poses were initialized randomly with their axes aligned with the axis of the implant’s screw hole. Each target point was flagged as successfully traversed whenever the tip position reached the 2-mm neighborhood of the point. The *ϵ* value for the RCM constraint (10) was chosen as 1 mm to account for the wiggle room between the CM and the implant’s screw hole. If before reaching a target point, the robotic system or rigid tool got to the cone VF boundary (infeasible optimization problem), the controller was switched to the secondary mode to recover from the infeasible situation. For each target point, the controller or rigid tool was allowed to switch to secondary control mode at most three times, otherwise the target point was flagged as unreachable. With a 30° maximum deviation angle, the robotic system and rigid tool covered 91% and 54% of the region behind the acetabular component, whereas with a 45° maximum deviation angle, 98% and 71% of the region were covered using each approach, respectively (see Table I).

### B. Dexterity and Manipulability

Conventionally, Yoshikawa’s manipulability index [49] has been used as a measure for dexterity of manipulators

(7) $$\omega (q)=\sqrt{\text{det}\left(J(q)J^{T}(q)\right)}$$

where *J*(*q*) is the unconstrained Jacobian of the manipulator as a function of joints’ state *q*. This measure, however, cannot be directly applied to our system when it is subject to the RCM constraint. Sadeghian *et al*. [50] previously derived the constrained Jacobian and manipulability index for systems subject to RCM constraint. The constrained Jacobian (*J*_*d*_) is formulated as

(8) $$J_{d}=J_{s}\;T\left[\underset{A}{\underset{︸}{\overset{I_{n-3}}{-J_{\text{RCM},II}^{-1}\cdot J_{\text{RCM},I}}}}\right]$$

where *T* is a permutation matrix to rearrange the system’s joints order such that $J_{\text{RCM},II}\in {\mathbb{R}}^{3\times 3}$ is invertible, *I* is the identity matrix, and *J*_*s*_ is the system Jacobian defined in (1). Furthermore, the constrained manipulability ellipsoid and measure could be formulated as [50]

(9) $${\dot{x}}_{\text{tip }}^{T}\underset{B}{\underset{︸}{{\left(J_{d}J_{d}^{T}\right)}^{-1}J_{d}\left(I+A^{T}A\right)J_{d}^{T}{\left(J_{d}J_{d}^{T}\right)}^{-1}}}{\dot{x}}_{\text{tip }}=1\;\omega (q)=\frac{\left|\text{det}\left(J_{d}J_{d}^{T}\right)\right|}{\sqrt{\text{det}\left(J_{d}\left(I+A^{T}A\right)J_{d}^{T}\right)}}$$

where the eigenvectors of *B* define the principal axes of the ellipsoid and the eigenvalues of *B* are the reciprocals of the squares of the ellipsoid’s semiaxes, *a*^−2^, *b*^−2^, and *c*^−2^. The manipulability ellipsoid volume is subsequently computed by $v=\frac{4}{3}\pi abc$.

**Fig. 12.**
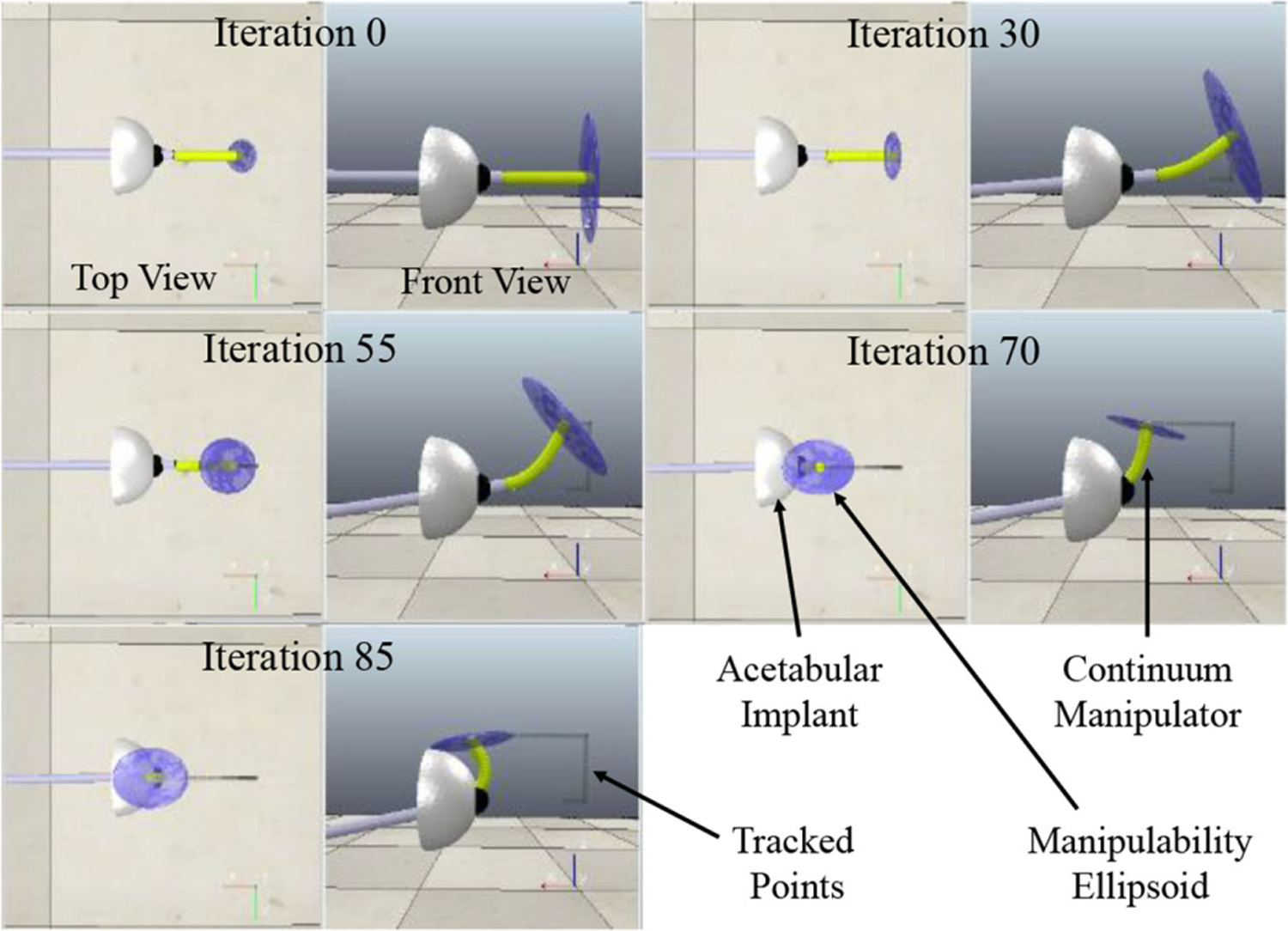
Snapshots of the evolution of the manipulability ellipsoid using the proposed robotic system.

To measure the constrained dexterity and manipulability of our system in confined spaces, we performed experiments in simulation where the system was concurrently controlled behind the acetabular implant while the RCM constraint was active. A set of target goal points were passed to the controller such so that the system would pass through the immediate points behind the implant. The goal of this experiment was to demonstrate how the constrained manipulability ellipsoid (9) evolves over time as the system’s tip approaches the vicinity of the implant. The constrained manipulability measure and the ellipsoid volume were compared for the combined system (UR-10 and CM) and only the UR-10 end-effector shaft without the CM. Fig. 12 shows the evolution of the constrained manipulability ellipsoid for the combined system as the system approaches the difficult-to-reach points behind the implant. Table II summarizes the results for the constrained manipulability index (9) for the combined system as well as the UR-10 only without the CM. The table shows that the manipulability index of the combined system is 12 times that of just the robot.

### C. Constrained Controller

In practice, the optimization problems (1), (4), and (18) are special cases of the general quadratic programming problems with sum of two-norms in the objective and linear or nonlinear constraints. Sequential quadratic programming or convex optimization solvers could be employed to solve these problems [51]. We used the methods provided in [52] and [53] with C++ and Python open-source implementations available freely.^1, 2^

The concurrent constrained control framework was implemented in simulation (Python) and on real hardware (C++). Different combinations of objectives and constraints were tested and evaluated. Moreover, the best combination of constraints in a practical surgical scenario was studied and strategies for avoiding or recovering from an infeasible optimization problem were provided. The capability of the system in maintaining the constraints was studied and the best parameters for tuning the optimization framework were presented. To fully understand the CM behavior when interacting with obstacles, experiments were performed to evaluate the requirement and efficacy of an adaptive Jacobian estimation approach (18) for the CM compared to a fixed predetermined Jacobian.

**Fig. 13.**
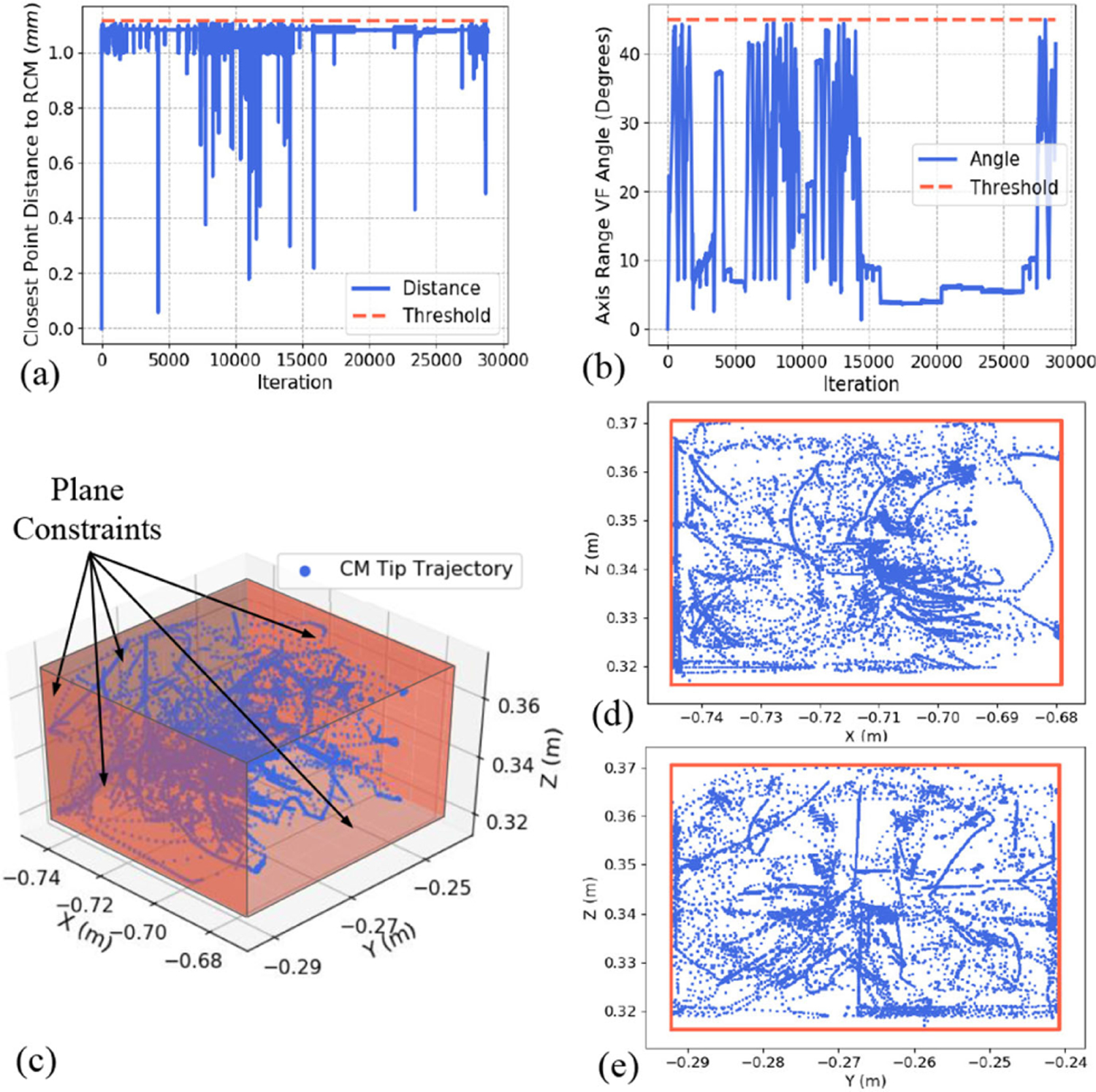
Performance of the controller in maintaining the constraints. (a) RCM VF. (b) Axis range VF angle. (c) Plane constraints bounding the CM’s tip position. Projection of the plane constraints in (d) *X-Z* and (e) *Y-Z* planes.

The constraints mentioned in Section IV were tested in execution of different surgical plans in reaching desired surgical points behind the acetabular cup implant. Results for maintaining RCM, axis range VF, hyperplane VF, and velocity and joint limit constraints are presented during exploration of the workspace behind the implant. Fig. 13(a) shows the distance between the desired programmed RCM point and the closest point to the RCM on the robot’s end-effector shaft (10). As observed, this distance remains below the specified threshold throughout the execution of the experiment. Fig. 13(b) demonstrates the angle between the robot’s end-effector shaft and the implant’s screw hole axis, which stays below the specified threshold of 45°. Fig. 13(c)–(e) displays the path traversed by the tip of the CM bounded by the five hyperplane constraints in 3-D and projected views. Table III summarizes the used joint velocity and position limits.

**Fig. 14.**
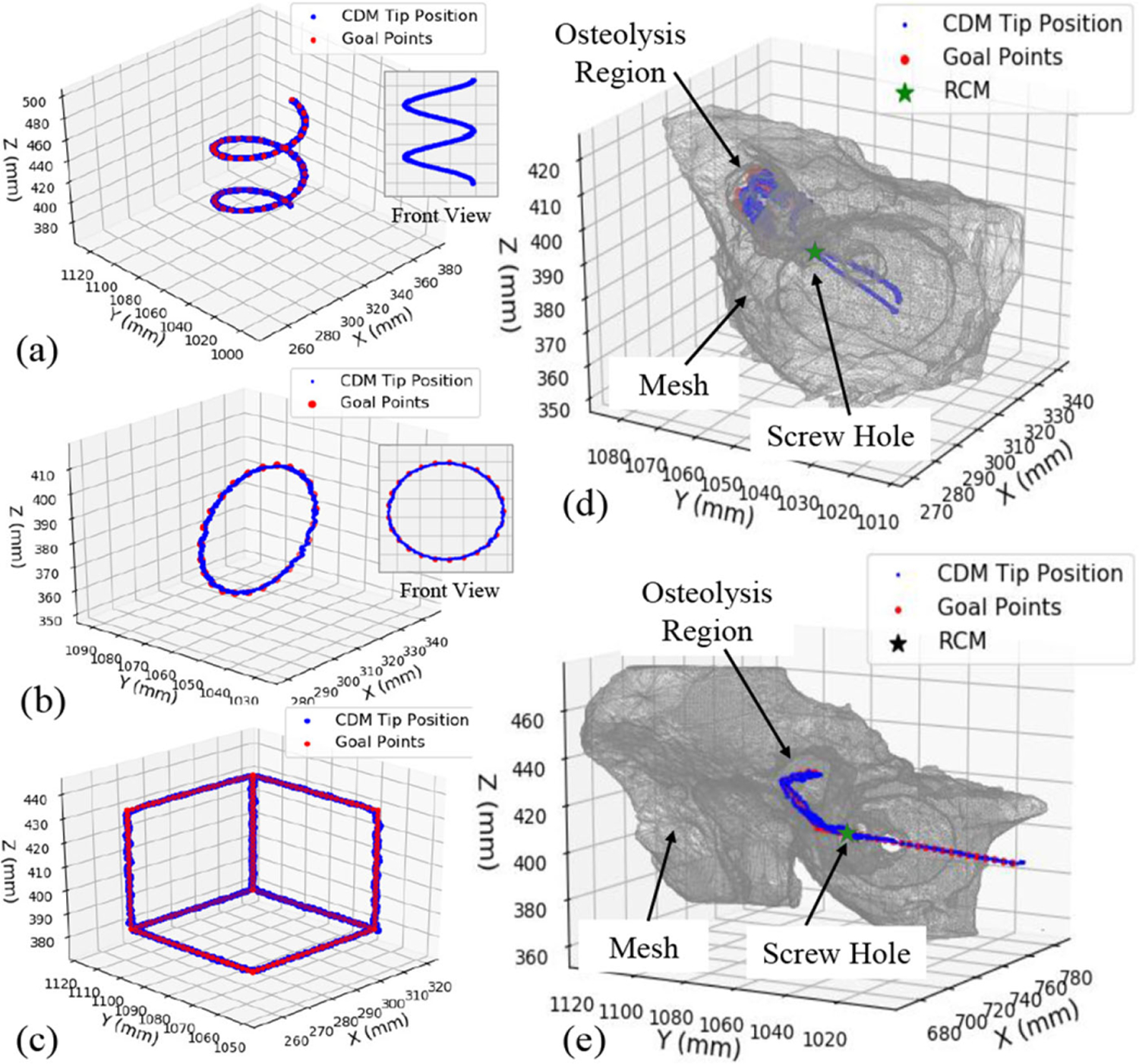
Controller performance in executing various motions and surgical plans. (a) Spiral motion. (b) Circle. (c) Cube. (d) Tracing the surface of the outlined osteolysis lesion on the preoperative CT. (e) Executing the surgical plan during debridement of hard sawbone phantom.

The PD controller parameters were determined experimentally as *k*_*p*_ = 0.4 and *k*_*d*_ = 0.1 to avoid possible overshoot. Sample trajectory tracking scenarios with these control parameters are demonstrated in Fig. 14. In particular, the controller was tested during path following of a spiral [see Fig. 14(a)], a circle [see Fig. 14(b)], a cube [see Fig. 14(c)], a surgical plan tracing the entire surface of the osteolysis lesion cavity outlined in the preoperative CT [see Fig. 14(d)], and a surgical plan for surface debridement of hard sawbone phantom behind the acetabular implant [see Fig. 14(e)]. Using a goal reaching threshold of 1.5 mm, the mean, standard deviation, and maximum error in tracking accuracy for these experiments were 1.47, 0.02, and 1.49 mm, respectively.

**Fig. 15.**
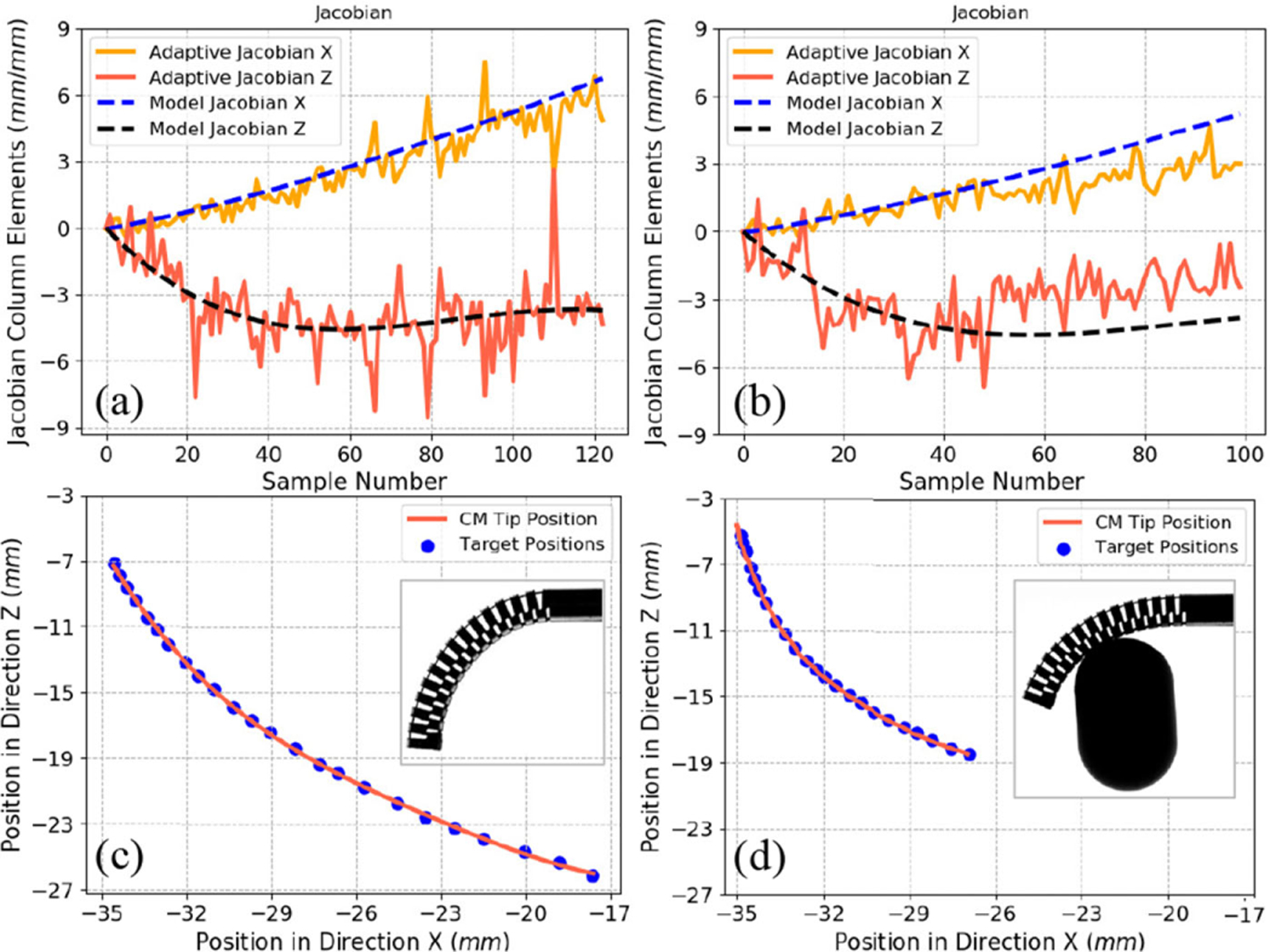
CMJacobian (*J*_*C*_) components using themodel-based and model-less estimation approaches in (a) free environment and (b) interaction with obstacle. Path following using the model-based Jacobian in (c) free environment and (d) interaction with obstacle.

The fixed model-based Jacobian obtained from derivation of the CM kinematics was also compared with the adaptive (model-less) Jacobian approach (18), where the CM Jacobian was updated on-the-fly during the experiment. To fully study the tradeoff between the two approaches, we performed two sets of experiments where the CM is commanded to a desired goal tip position in free environment while it collides with an obstacle. In these experiments, we first obtained the fixed model-based CM Jacobian in free environment bending by recording the tip position and cable tension and fitting a Bernstein polynomial to the collected data. The tip position was collected in a similar fashion, as outlined in Section IV–C, using a custom-designed optical tracker reflective geometry mounted on the CM distal end. In the first part of each experiment, the model-based Jacobian was used and cable displacement commands were generated by (1) while the joint position and velocity constraints were applied to avoid overtension of the cable or damage to the CM. The second part of each experiment was composed of initializing the CM Jacobian with the model-based Jacobian and then iteratively updating it via (18) through the rest of the experiments with ϒ = 1 while generating cable displacements using (1). Results are demonstrated in Fig. 15(a) and (b), where the components of the Jacobian column associated with the CM actuation cable are plotted for the free environment and obstacle interaction experiments, respectively. The path tracking for each of these experiments is plotted in Fig. 15(c) and (d), where the recorded CM tip positions follow the target points on the desired path.

### D. Sensing

The system’s sensing module was evaluated against ground-truth data in several experiments. This was done in two separate steps: First, evaluating the performance of the FBG sensor to estimate the tip position of the CM both in free and constrained environments where the CM was interacting with obstacles at various locations along its body, and second, evaluating the overall system tip position with respect to the base of the UR-10 by combining information from the FBG sensor and the optical tracker reflective jig 2. As outlined in Section IV–C, the ground truth data for the tip position was obtained by mounting an optical tracker jig on the CM distal end (jig 1 in Fig. 6). It must be noted that the hand-eye calibration procedure (see Section VI–A) was implicitly assessed in the aforementioned evaluation process, as it was the bridge between the optical tracker (end-effector shaft pose) and FBG data (CM shape).

**Fig. 16.**
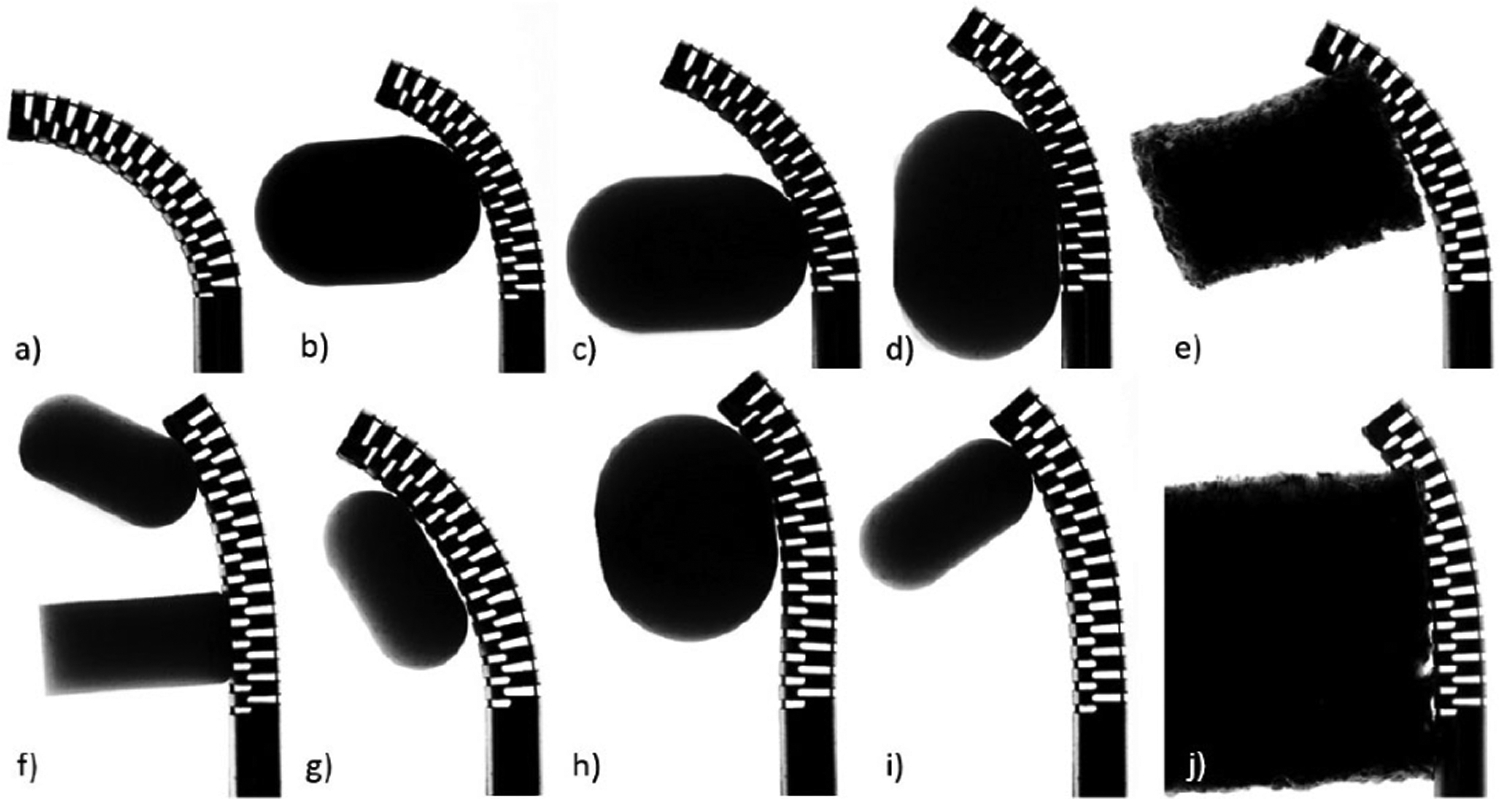
Experiments. (a) CM bending in free space. (e) and (j) colliding with soft obstacle, and (b), (c), (d), (f), (g), (h), and (i), colliding with hard obstacles.

To evaluate the accuracy of the FBG sensor in estimating the CM tip position, a total of 19 experiments were carried out, 10 of which were in free environment and the other 9 included an obstacle located randomly along the length of the CM’s body (see Fig. 16). A total of 68 306 and 61 642 samples were collected in the free and obstacle experiments, respectively. The data-driven method outlined in Section IV–C was trained and tested against this dataset with *k*-fold cross-validation. The dataset was split randomly into 80% training and 20% testing data with respect to the number of overall experiment sets and the trained model was tested against the testing dataset (*k* = 5). The tip position estimation error mean and standard deviation of all the five-fold split combinations were 0.37 and 0.12 mm, respectively. In the worst case, the mean, standard deviation, and maximum tip position error were 0.58, 0.27, and 0.88 mm, respectively.

The tip position estimation obtained from the FBG sensor was expressed with respect to the base of the CM. Combining this information with the end-effector shaft pose obtained from the optical tracker reflective jig2 (see Fig. 6) and the transformations obtained from the hand-eye calibration, the CM tip position can be expressed with respect to the base of the entire robotic system and used as feedback in the controller framework (see Section IV). To evaluate the accuracy of the entire system tip position estimation, the CM and the positioning robot were moved concurrently in the vicinity of the desired workspace and ground-truth data were obtained by direct sensing of the CM tip position with respect to the base of the robot. The mean and standard deviation of the entire system tip tracking error were 0.50 and 0.18 mm, respectively, with maximum error of 1.46 mm. Such precision in TPE for the entire robotic system provided accurate real-time feedback to the controller when bringing the system tip position to the desired surgical target points.

### E. Planning and Debridement Performance

As part of the planning, the registration of the preoperative CT to the phantom and the human cadaver must be performed. To do so, as outlined in Section VI–B, the surface of the 3-D printed model in the phantom study and the acetabular implant and the surrounding bone in the human cadaver study were digitized using an optical tracker digitization tool (see Fig. 9). The registration process (6) was then performed and consequently, any planning on the preoperative CT could be expressed in the robot base coordinate system. With the use of the flexible debridement instruments and the concurrent control of the developed system, we then demonstrated that it is possible to perform drilling and milling both on simulated sawbone phantom and human cadaver hard bone in confined spaces.

As outlined in Section VI–B, in the first step of the registration, four points on the screw holes of the implant were chosen as fiducials for finding an initial registration guess [green points in Fig. 9(b)]. The root mean square for this step of the registration was 1.80 mm. In the second step, several points were digitized on the surface of the acetabular cup implant and the surrounding exposed bone (overall 76 points). Using this point cloud, the second step of the registration (point cloud to surface registration) was completed and the mean, min, and max residual errors for this step were measured as 0.87, 0.02, and 3.50 mm, respectively. Fig. 9 shows the overlay of the digitized points on the preoperative CT after registration (red points).

**Fig. 17.**
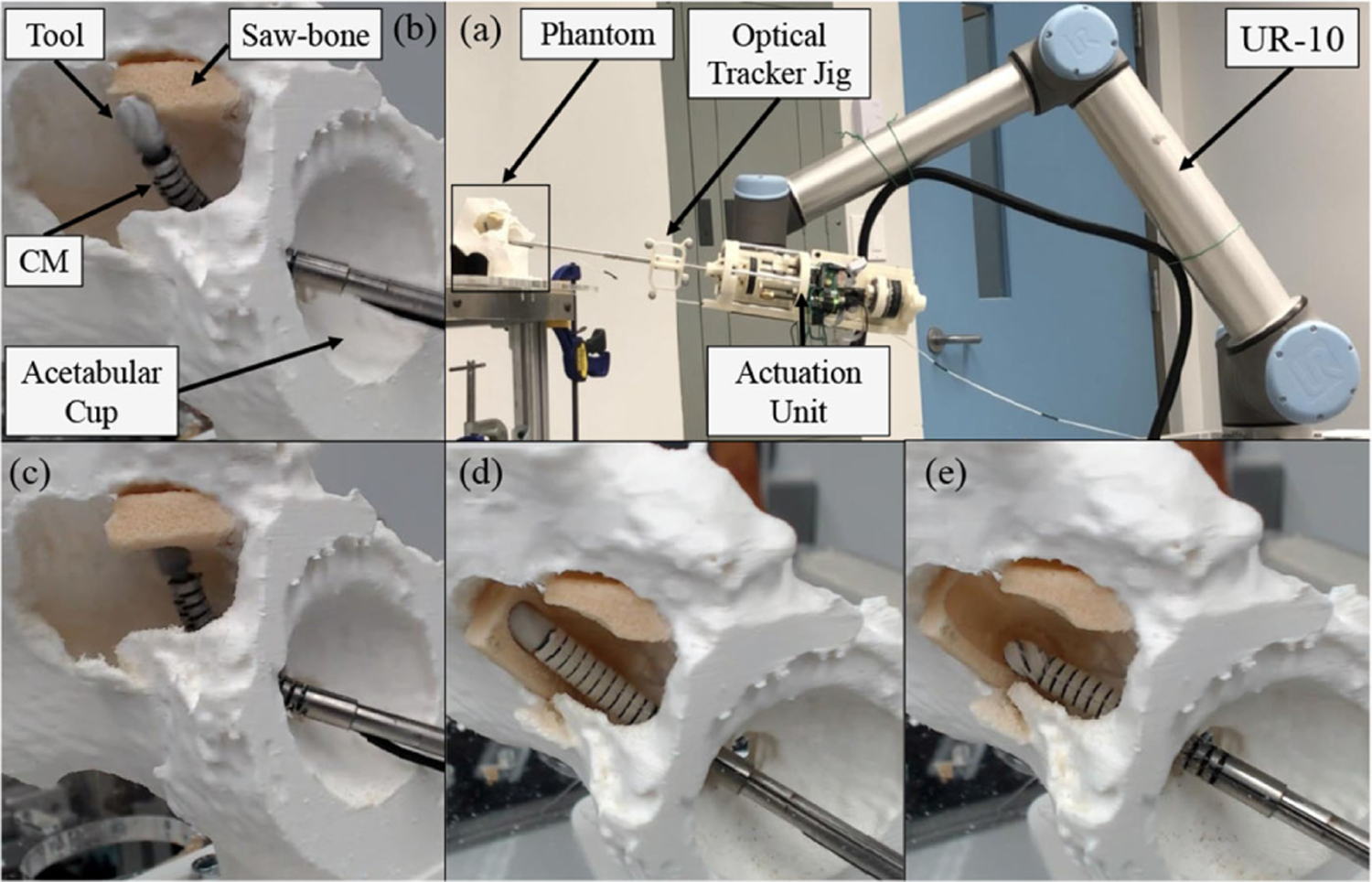
Concurrent control of the robotic system during debridement tasks in phantom studies. (a) Experimental setup and the robotic system. (b)–(e) Various successful surface debridement tasks on sawbone phantoms mounted behind the acetabular implant.

**Fig. 18.**
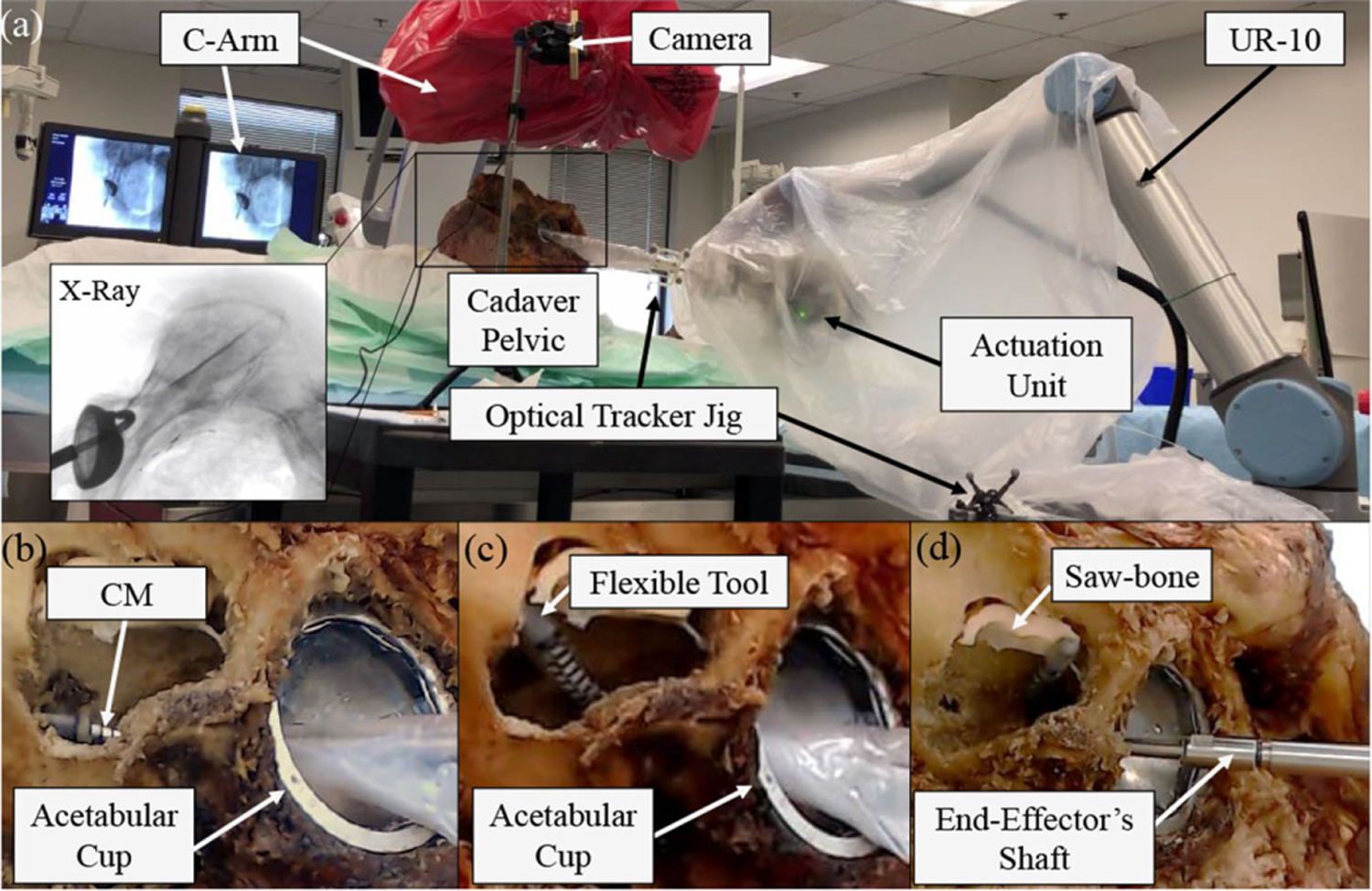
Concurrent control of the robotic system during debridement tasks in cadaver studies. (a) Robotic system deployed in the operating room. (b) Drilling task inside hard bone. (c) Surface milling task of hard bone. (d) Reaching extremely difficult points right behind the implant while milling hard sawbone phantoms.

Given the registration, any set of desired target points on the patient CT can be transformed to the coordinate system of the robotic system base. To demonstrate the capability of the system in autonomous debridement of simulated phantom and bone lesions, we outlined and executed several surgical paths both in phantom and cadaver studies. Fig. 17 shows the surface milling capabilities of the system in various experiments with the hard sawbone phantom mounted to the back and top of the cavity. Similarly, in the cadaver study, drilling and milling of hard bone are demonstrated in Fig. 18 with the system of UR-10 and CM concurrently controlled to the surgical target points. To make the debridement task further challenging, hard simulated phantom was mounted at difficult-to-reach locations right behind and above the acetabular cup implant, where the lesion area was outlined by our clinical collaborator [see Fig. 18(d)] and the system successfully reached this area while debriding the hard sawbone. Similar to the controller validation experiments and using a goal reaching threshold of 1.5 mm, the mean, standard deviation, and maximum error in tracking accuracy for these experiments were 1.47, 0.02, and 1.50 mm, respectively. During the debridement tasks, the flexible instrument’s rotation velocity was set at 3200 r/min (see the works in [34] and [35] for further details on the instrument velocity during cutting tasks).

## IX. Discussion

Autonomous surgical systems, such as ROBODOC, have been deployed in the operating rooms for years in orthopedic applications, yet the incorporation of rigid-link robots as their core system component has been a limitation for adaptation to confined anatomies. The proposed system in this article could overcome this limitation by incorporation of a CM that is well suited for orthopedic applications, which enhances dexterity and patient access. Additionally, the current paradigm of robot-assisted surgeries depends mostly on an individual surgeon’s manual capability. Autonomous robotic surgery, on the other hand, promises enhanced efficacy, safety, and improved surgical outcomes under the surgeon guidance. Through human cadaver experiment, we demonstrated the feasibility of using a dexterous autonomous system with task autonomy not only to reach difficult-to-access locations in human anatomy, but to successfully mill hard bone as well. It is worthwhile to mention, however, that in our system, the surgeons are still in full control and supervision of the surgery, i.e., they indicate the surgical plan and they monitor and intervene during the surgery, as needed.

The constrained workspace analysis results show that our developed system out-performs the conventional rigid tools, such as curettes in confined spaces in human anatomies, where the rigid tools cannot maneuver as much. This is achieved by the flexibility and extreme bending capabilities of our developed CM. For the case of pelvic osteolysis and when restricting the axis range limit to 45°, the combined robotic system can achieve 98% of the lesion area behind the acetabular implant, whereas a rigid tool can at most reach 71% of this area, excluding the most important and problematic locations that are right behind the implant. This limitation worsens for the rigid tool (54% coverage) if the axis range of motion is restricted further to 30°, whereas the robotic system can still cover 91% of the lesion area. The importance of this extended dexterity by the robotic system is realized further in the context of MIS, where a more restricted axis range limit requires a smaller incision on the patient while most of the lesion area is still reachable. As demonstrated in Fig. 11(a), our developed system can reach the surface behind the implant even by only bending the CM while the robot shaft is fixated and aligned with the axis of the implant’s screw hole. This can be regarded as a significant advantage of using the robotic system over rigid tools to perform the surgery in a minimally invasive fashion with a small incision point on the patient’s skin.

The evolution of the manipulability ellipsoid in Fig. 12 reveals that the addition of the CM to the system enhances the constrained manipulability in two ways. Explicitly, the extreme articulation capabilities of the CM increases the manipulability in CM’s direction of bending. This can be observed in the front view images where the direction of maximum manipulability is aligned with the direction of CM bending. Implicitly, when the CM is bent, additional manipulability is achieved by the actuation unit’s roll motor (roll about the end-effector shaft) in the direction perpendicular to the CM’s plane of bend. This can be viewed on the top view images where in the earlier iterations (straight CM), the semiaxis of the ellipsoid is relatively small in this direction, whereas as the CM bends more toward the later iterations, the semiaxis in the direction perpendicular to the CM’s plane of bend enlarges. The constrained manipulability index is compared in Table II where the mean manipulability index for the combined system is nearly 10^12^ times more than the robotic system alone when the RCM constraint is active.

**Fig. 19.**
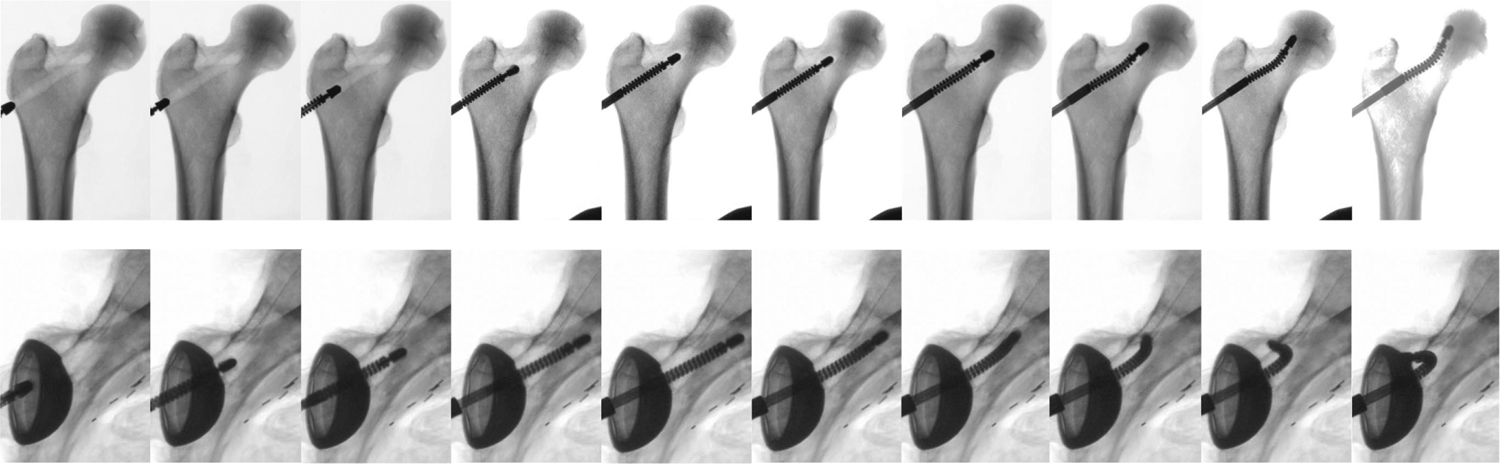
X-ray snapshots of two cadaver experiments demonstrating extreme reach and dexterity of the developed system for top row: core decompression of the femoral head osteonecrosis using the curved-drilling technique, and bottom row: less-invasive treatment of pelvic osteolysis behind the acetabular implant.

In addition to the less invasive treatment of pelvic osteolysis behind the acetabular component, we demonstrate how our developed system could be used for core decompression of the femoral head osteonecrosis using the curved drilling technique [35]. In this procedure, the UR-10’s forward and CM’s bending motions are combined to drill curved branches inside the confined femoral head to enhance the reach to osteonecrotic bone, not readily accessible by conventional drills and rigid instruments. The combination of the CM cable actuation velocity and forward feeding rate of the UR-10 determines the profile of the curved-drilled tunnel. An advantage of using the developed system is that multiple velocity combinations of the robotic system could be performed to achieve multiple branches inside the bone to further increase lesion removal compared to conventional rigid tools. Fig. 19 top row demonstrates X-ray snapshots of an example curved-drilled tunnel in femur head, whereas the bottom row shows the less invasive treatment of pelvic osteolysis. The extreme reach and dexterity of our developed system in confined spaces in human anatomy can be perceived over conventional rigid tools.

The constrained controller results demonstrate that the optimization framework is successful in maintaining the desired constraints while executing various surgical tasks with great target placement accuracy. It should be noted that the threshold specified for reaching the goal points was set to 1.5 mm during the experiments, which is satisfactory for orthopedic applications, considering the scales and dimensions of the region of interest. Depending on the application and requirements, this value is adjustable and smaller thresholds could be imposed on the controller in favor of further accuracy at the expense of slightly longer convergence time. For the joint position and velocity constraints, a tradeoff between system safety, time, and the extent of reach exists. For instance, in the simulation environment, we allowed the robotic system to move slightly faster and with greater range compared to the physical system since there is no concern of damage to the system or patient in simulation. From a surgical standpoint, however, to avoid possible bone necrosis caused by the feeding rate of the tool, and considering the safety of the surgical staff, the patient, and the robotic system, extra care must be taken into account.

Another important safety aspect is the detection of CM contacts with the anatomy and determination of safe or unprecedented interactions. Previous work [40] proposed a learning-based framework to detect CM contacts with the environment using only the FBG sensor. This method could be further extended by incorporation of additional contact/force sensors to fully determine and measure contact forces. The constrained controller could then account for the dynamics in addition to robot kinematics to complement a safe operation.

In recent years, there has been an emerging interest on incorporation of the model-less Jacobian approach to estimate the CM Jacobian on-the-fly during control [54], [55]. While potentially beneficial, the tradeoffs in using this approach must be well studied and realized in practice. As observed in Fig. 15(a), the model-based Jacobian obtained experimentally and the computed model-less Jacobian follow a similar trend in free environment motion, as expected. When the CM interacts with obstacles [see Fig. 15(b)], the two approaches still exhibit a similar behavior, although the magnitude of the Jacobian columns in the model-based approach become slightly inaccurate compared to the estimated Jacobian from the model-less approach. The target points are, however, reached successfully despite the interaction with the obstacle and the slightly inaccurate CM Jacobian. This can be justified by noting that the most important factor when generating joint commands using (1) is if the Jacobian direction is accurate. In other words, as long as the model-based Jacobian is functional-enough in the correct direction, the generated commands will lead the CM toward the goal. The model-less Jacobian approach, on the other hand, generates noisy estimations as expected from the numerical methods. This noise can be problematic in certain cases, for instance when the CM is close to its straight pose. As observed in Fig. 15(a) and (b), before nearly iteration 20 (when the CM is straight), the estimated Jacobian values from the model-less approach sometimes generates values with incorrect sign (direction) caused by numerical errors. Consequently, we used the model-based approach for robot control in the phantom and cadaver experiments.

While in this work, machine learning techniques were incorporated in the FBG sensing component of the system, these techniques could be extended to other components of the surgical system, such as the planning module. For instance in orthopedics, researchers have recently incorporated deep learning techniques for automated detection and classification of knee arthroplasty [56].

The submillimeter sensing and control tracking accuracy demonstrated in this work improves the accuracy achieved by manual tools conventionally used by surgeons. The kinematic redundancy introduced by the 9-DOF robotic system allows for incorporation of various surgical constraints into the controller while executing desired surgical plans. The maximum 198° planar bend of the CM [18], combined with the robot manipulator motion results in 98% coverage behind the acetabular implant, which is far superior than the 54% achieved by conventional rigid surgical tools. The system is designed such that only the CM, embedded flexible tools, and FBG sensor are exposed to the anatomy during surgery and all other components are entirely covered and sealed. The exposed components, therefore, need to be carefully sterilized before the surgery. After the surgery, the flexible tool is released from the actuation unit by the quick connection mechanism and the CM and tool are removed from the distal end of the unit.

## X. Conclusion

This work was the first comprehensive human cadaver study demonstrating extreme dexterity, improved patient access, and hard bone debridement capabilities that could be achieved by CMs for less and minimally invasive orthopedic interventions. The developed system enhanced the reach and workspace coverage in surgical applications where conventional rigid instruments performed suboptimal. Robot-assisted less-invasive treatment of hip osteolysis as well as the curved drilling technique for core decompression of femoral head osteonecrosis were demonstrated as two immediate orthopedic applications that could benefit from flexibility and dexterity of the developed system. Future work will emphasize on design improvements and analysis of sterilization and deployment of the system in the operating room.

While the primary envisioned use case of the system was minimally invasive orthopedic interventions, the enhanced dexterity of the system could potentially benefit other surgical applications, such as spine and otorhinolaryngology. For potential applications at other scales, the CM dimensions and the choice of the positioning robot can be adjusted accordingly in the next generations of the system. While full surgical autonomy still remains in the realm of science fiction, pushing the technological potentials to the boundaries while realizing a meaningful collaboration and team work between the surgeon and robotic systems could greatly benefit the future of healthcare.

## Figures and Tables

**Fig. 5.**
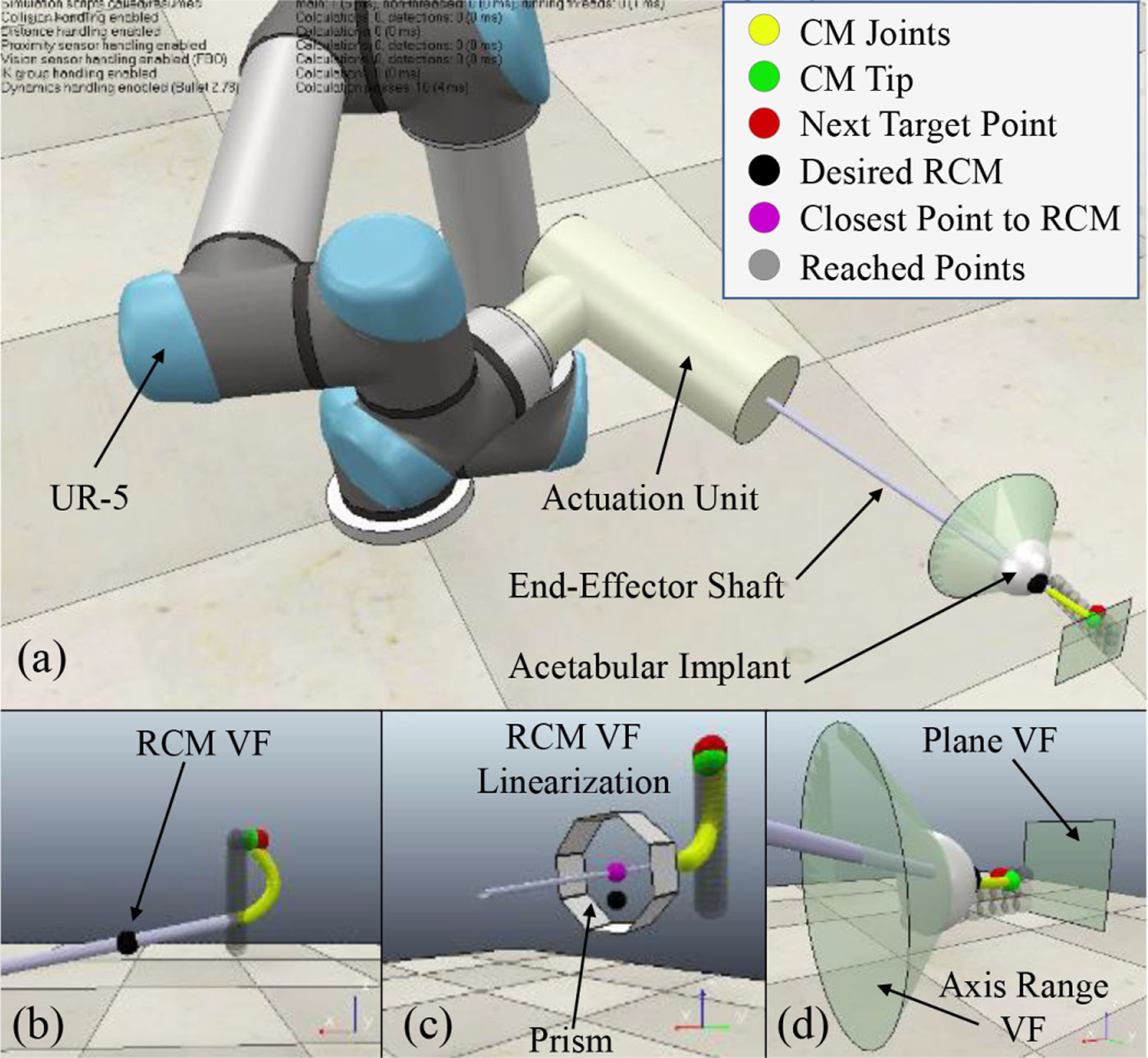
Developed simulation environment with (a) accurate models of the positioning robot, the actuation unit, the acetabular implant, and the CM. (b) Robotic system satisfying the RCM constraint. (c) Linearized RCM constraint using a prism with eight faces and the closest point to the RCM on the end-effector shaft. (d) Axis range and plane VF.

**TABLE I:** Comparison of the Workspace Coverage Behind the Implant for Robotic System and Rigid Tool Subject to Axis Range VF

	Robotic System	Rigid Tool
Axis Range VF (30)	91%	54%
Axis Range VF (45)	98%	71%

**TABLE II:** Constrained Manipulability Index Comparison

	Constrained Manipulability Index			
	Mean	Std. Dev.	Max	Min
UR Only	8.42 e-17	1.63 e-16	7.53 e-16	1.23 e-50
UR + CM	1.31 e-05	1.28 e-05	4.29 e-05	1.85 e-07

**TABLE III:** Comparison of Joint Limits in Simulation and Hardware

		UR Joints	Roll	CM
Position Limit	Sim.	±2*π* (rad)	±2*π* (rad)	0–10 (mm)
	Hardware	±2*π* (rad)	±2*π* (rad)	0–7 (mm)
Velocity Limit	Sim.	±5e-3 (rad/s)	±5e-2 (rad/s)	2e-1 (mm/s)
	Hardware	±3e-3 (rad/s)	±3e-3 (rad/s)	5e-1 (mm/s)

## Footnotes

1
www.netlib.org

2
www.cvxpy.org

## Appendix A Control Framework Constraints

### A. Programmable RCM

A programmable RCM [57] is a versatile software approach for systems that do not contain a mechanical RCM. A great advantage is that the desired RCM can be readily set in the software and the robotic system maintains the constraint without incorporation of any special mechanical design. The constraint can be derived by restricting the point on the robot that is closest to the programmed RCM to stay in the vicinity of the RCM bounded by a circle with radius *ϵ*. For ease of computation, this nonlinear constraint can be approximated by linearization to maintain the robot point within a prism with *m* faces (see Fig. 5). This can be formulated as *m* inequality constraints in (1), where

(10)
$$\begin{array}{l} H_{i}=v_{i}^{T}J_{\text{RCM}}^{v}\\ h_{i}=\epsilon +v_{i}^{T}\left(p_{\text{RCM}}-p_{\text{closest }}^{k}\right) \end{array}\}\text{ for }i=1,\ldots ,m$$

where $J_{\text{RCM}}^{v}$ corresponds to the linear component of the Jacobian resolved at a point on the robotic system that is closest to the RCM at time step *k* and *v*_*i*_’s are the normals to the faces of the prism. Of note, the tradeoff between run-time and accuracy of the linear approximation for this constraint can be realized by the appropriate choice of *m*. We use eight faces in our experiments for good accuracy and fast performance [51].

### B. Axis Range VF

To increase patient and system safety, the range of motion of the robotic system end-effector shaft must be restricted. A conical VF can be defined to limit the axis of the robotic system to always stay within a cone with the set RCM as its apex, tapered along a programmable desired axis (*d*_des_) with maximum allowable deviation *ϕ*_*a*_. At time step *k*, this VF can be formulated as a single inequality constraint in (1), where

(11)
$$H=-d_{\text{des}}^{T}J_{\text{sh}}^{\omega }\;h=d_{\text{des}}^{T}d_{\text{sh}}^{k}-\text{cos}\left(\phi_{a}\right)$$

where $d_{\text{sh}}^{k}$ refers to the direction of the end-effector shaft at time step *k*, and $J_{\text{sh}}^{\omega }$ is the angular component of the Jacobian resolved at any point on the shaft (e.g., CM base).

### C. Hyperplane VF

Another useful constraint is to prohibit a particular point on the robotic system to pass a desired plane. An example of such scenario arises when a sensitive region in the anatomy should be avoided by the instrument. Another use case of this VF is to limit the amount that the end-effector shaft can be inserted into the anatomy. Assuming the desired hyperplane is defined as *ax* + *by* + *cz* = *t*, with the normal *n* = [*a b c*]^*T*^, the constraint can be written as a single inequality constraint in (1), where

(12)
$$H=-n^{T}J_{p}^{\omega }\;h=n^{T}p_{p}^{k}-t$$

where the subscript *p* refers to the desired point on the system (e.g., the instrument tip or the CM base) that must avoid entering a particular region. Of note, a multitude of these hyperplanes could be incorporated as inequality constraints to define a forbidden (or allowable) region.

### D. Velocity Constraints

To make sure that the system runs at speeds that are both safe and beneficial for cutting but also efficient, velocity constraints both in configuration and task-space could be added to the optimization problem

(13)
$$H={\left[-I_{9\times 9}\;I_{9\times 9}\;-J_{s}^{T}\;J_{s}^{T}\right]}^{T}\;h={\left[-q_{v,l}^{T}\;q_{v,u}^{T}\;-\xi_{v,l}^{T}\;\xi_{v,u}^{T}\right]}^{T}$$

where the lower and upper joint and task-space velocity bound vectors *q*_*v*,*l*_, *q*_*v*,*u*_, *ξ*_*v*,*l*_, and *ξ*_*v*,*u*_ respectively, are experimentally determined by assessing safety constraints as well as smoothness of operation across test runs of the system.

### E. Joint Limit Constraints

To ensure safe manipulation of the system, joints positions are constrained to *q*_*p*,*l*_ and *q*_*p*,*u*_ to avoid potential damage to the system due to collision, actuation cable damage, etc. These constraints can be formulated as

(14)
$$H={\left[-I_{9\times 9}\;I_{9\times 9}\right]}^{T}\;h=\left[q-q_{p,l}\;-q+q_{p,u}\right].$$

## Appendix B Control Framework Regularization

### A. Redundancy Resolution

The redundancy in the developed system (9 DOF) can be resolved at the velocity level by introducing a regularization term in the form of *R*(Δ*q*) = ∥Δ*q*∥_Γ_ = Δ*q*^*T*^ΓΔ*q*. Presumably any joint is prohibited from moving too fast, resulting in a more controllable motion and increased safety. Additionally, the redundancy available beyond that required for the CM tip motion may be freely used to assist in the realization of some chosen objective. For instance, the weight matrix Γ can be chosen such that for execution of a particular task, specific system joints (e.g., CM joints) are employed more than the rest. This can be achieved by applying a larger penalty (weight) to the joints that should not move as much.

### B. Stay Near Axis

The axis range VF mentioned earlier merely restricts the end-effector shaft to remain within a desired conical region. In particular surgical scenarios, however, it may be useful to enforce the end-effector shaft to stay near a desired axis. This can be added to the objective function of (1) as a regularization term formulated by

(15)
$$\gamma {\left\|d_{\text{des}}^{T}\left(J_{\text{sh}}^{\omega }\Delta q+d_{\text{sh}}^{k}\right)-1\right\|}_{2}$$

where *γ* is the damping factor and the goal is to generate configuration space commands at step *k* that drive the end-effector shaft axis closer to a desired axis. In another scenario, (15) can be used as the main objective function to recover from an infeasible optimization problem, as will be described in Appendix B–D.

### C. Stay Near Pose

As another recovery strategy from infeasible optimization problem, it might be desired to bring the system to the proximity of a desired configuration (*q*_des_). Given the configuration of the system at time step *k* (*q*^*k*^), this can be formulated with a damping factor (*η*) in the objective function as

(16)
$$\eta {\left\|\Delta q-\left(q_{\text{des}}-q^{k}\right)\right\|}_{2}.$$

### D. Infeasible Problem Recovery Strategy

A common occurring scenario with activated axis range VF is that the optimization problem (1) inevitably becomes infeasible when the end-effector shaft axis approaches the boundaries of the cone VF. As such, a recovering strategy must be deployed to drive the system back to ward feasible region while still maintaining the constraints, such as the RCM. A reasonable strategy is to switch to a secondary control mode where the goal is to escape the infeasible region by bringing the end-effector shaft toward the center of the cone using (15) or bringing the system toward the initial configuration using (16). The secondary control mode is achieved by setting either *γ* = 1 or *η* = 1 in (15) and (16) and setting *α* = 0 in (1) while maintaining the *H* and *h* constraints. As soon as the end-effector shaft axis was sufficiently far from the cone boundaries (by measuring the angle from the screw hole axis), the controller was switched back to the primary mode (*α* = 1, *η* = 0, *γ* = 0). The recovery strategy ensures the system is brought to a configuration far from the constraints boundaries such that the feasible region for the optimization-based controller is increased. Consequently, the controller can generate motion commands that satisfy the constraints far from the bounds to continue the execution of the surgical plan. In this stage, the constraints could also be optionally adjusted (e.g., through penalizing terms in the regularization terms of the optimization), to ensure future generated commands will avoid going to configurations close to constraint boundaries.

## Appendix C CM Jacobian

The CM Jacobian could found by fitting a model to empirical data obtained from the actuation cable displacements (*l*_*c*_) and an overhead camera tracking the CM tip (*p*_cam_ = [*p*_*x*_ *p*_*y*_]). Previous investigation of the collected data [58] suggested a sinusoidal relationship between the two components of the CM tip position. As such, we first related *l*_*c*_ to *p*_*x*_ using a Bernstein polynomial of degree 7 and subsequently related *p*_*x*_ and *p*_*y*_ with a sinusoidal mapping

(17)
$$\{{}_{p_{y}=\sum_{j=1}^{3}a_{j}\text{sin}\left(b_{j}p_{x}+c_{j}\right)}^{p_{x}=\sum_{i=0}^{7}\beta_{i}\left(\begin{array}{c} 7\\ i \end{array}\right){\hat{l}}_{c}^{i}{\left(1-{\hat{l}}_{c}\right)}^{7-i}}$$

where *β*_*i*_, *a*_*j*_, *b*_*j*_, and *c*_*j*_ are the mapping parameters found in a least square fashion using the training data (pairs of {*l*_*c*_, *p*_cam_}), and ${\hat{l}}_{c}=\left(l_{\text{max}}-l_{c}\right)/l_{\text{max}}$ is the normalized CM cable length with *l*_max_ as the maximum pull length of the cable. The linear component of CM Jacobian expressed in the CM base frame can be obtained by taking the derivative of *p* = [*p*_*x*_ *p*_*y*_ 0]^*T*^ with respect to the cable parameters ${\hat{l}}_{c}$. To express this Jacobian in the robot base coordinate frame, the rotation matrix between the base of the robot and the base of the CM $\left(R_{C}^{R}\right)$ is applied to this matrix to find $J_{c}^{v}\in {\mathbb{R}}^{3\times 2}$. Clearly, bending of the CM does not alter the orientation of the end-effector shaft and, therefore, $J_{c}^{\omega }$ is a 3 × 2 zero matrix.

An alternative approach is to obtain *J*_*c*_ in a *model-less* fashion as proposed in, e.g., [38] and [54], where the CM Jacobian is re-estimated at each time-step base on the generated commands and the resulting motion. Presumably, this method can be beneficial in a compliant system where the Jacobian may vary as the CM interacts with the environment. This can be formulated as a second optimization problem to re-estimate only the CM Jacobian (*J*_*c*_) after each run of (1)

(18)
$$\text{minimize }{\left\|\Delta J_{c}^{k}\right\|}_{F}\;\text{subject to }\Delta x_{c}^{k}=\left(J_{c}^{k}+\Upsilon \Delta J_{c}^{k}\right)\Delta l_{c}^{k}$$

where ϒ is a scaling factor to reduce numerical noise and $\Delta x_{C}^{k}=x_{C}^{k}-x_{C}^{k-1}$ is the resulting motion of the CM tip at time step *k* due to its own actuation commands $\left(\Delta l_{c}^{k}\right)$ computed from (1). Note that *x*_*C*_ is the CM tip position expressed in the robot base coordinate frame computed by $R_{C}^{R}\cdot p_{\text{fbg}}$ and $J_{c}^{\omega }=0_{3\times 2}$ as before. In practice, to solve (18), we stacked the elements of $\Delta J_{c}^{k}$ in a column vector and minimized the two-norm of this vector. The constraint was also simply rewritten as a linear equality constraint using this vector. The optimization problem can then be easily solved using sequential quadratic programming solvers.
